# Biodiversity Assessment of the Fishes of Saba Bank Atoll, Netherlands Antilles

**DOI:** 10.1371/journal.pone.0010676

**Published:** 2010-05-21

**Authors:** Jeffrey T. Williams, Kent E. Carpenter, James L. Van Tassell, Paul Hoetjes, Wes Toller, Peter Etnoyer, Michael Smith

**Affiliations:** 1 Fish Division, Department of Vertebrate Zoology, National Museum of Natural History, Smithsonian Institution, Suitland, Maryland, United States of America; 2 Department of Biological Sciences, Old Dominion University, Norfolk, Virginia, United States of America; 3 Department of Ichthyology, American Museum of Natural History, New York, New York, United States of America; 4 Department of Environment and Nature, Ministry of Public Health and Social Development, Curaçao, Netherlands Antilles; 5 Saba Conservation Foundation, Fort Bay, Saba, Netherlands Antilles; 6 Harte Research Institute, Texas A&M University-Corpus Christi, Corpus Christi, Texas, United States of America; 7 Center for Applied Biodiversity Science, Conservation International, Arlington, Virginia, United States of America; Smithsonian's National Zoological Park, United States of America

## Abstract

Biodiversity surveys were conducted on Saba Bank, Netherlands Antilles, to assess ichthyofaunal richness and to compare with published surveys of other Caribbean localities. The primary objective was to estimate the total species richness of the Saba Bank ichthyofauna. A variety of sampling techniques was utilized to survey the fish species of both the visually accessible megafauna and the camouflaged and small-sized species comprising the cryptic ichthyofauna.

Based on results presented herein, the number of species known on Saba Bank is increased from 42 previously known species to 270 species. Expected species-accumulation curves demonstrate that the current estimate of species richness of fishes for Saba Bank under represents the actual richness, and our knowledge of the ichthyofauna has not plateaued. The total expected fish-species richness may be somewhere between 320 and 411 species.

The Saba Bank ichthyofaunal assemblage is compared to fish assemblages found elsewhere in the Caribbean. Despite the absence of shallow or emergent shore habitats like mangroves, Saba Bank ranks as having the eighth highest ichthyofaunal richness of surveyed localities in the Greater Caribbean. Some degree of habitat heterogeneity was evident. Fore-reef, patch-reef, and lagoonal habitats were sampled. Fish assemblages were significantly different between habitats. Species richness was highest on the fore reef, but 11 species were found only at lagoonal sites.

A comprehensive, annotated list of the fishes currently known to occur on Saba Bank, Netherland Antilles, is provided and color photographs of freshly collected specimens are presented for 165 of the listed species of Saba Bank fishes to facilitate identification and taxonomic comparison with similar taxa at other localities. Coloration of some species is shown for the first time. Preliminary analysis indicates that at least six undescribed new species were collected during the survey and these are indicated in the annotated list.

## Introduction

Saba Bank is the largest atoll in the Atlantic Ocean Basin and one of the three largest atolls on earth [1]. Located in the Dutch Windward Islands about 250 km east of Puerto Rico, it is a flat-topped seamount rising 1800 m from the surrounding sea floor. Except for the fact that it does not break the water surface, Saba Bank is a classic atoll consisting of a submerged mountain crowned at the summit with a ring of actively growing coral reefs [2]. Saba Bank is relatively free of the problems that are degrading many Caribbean reef systems, and the few problems it faces include anchoring and abrasion by oil tankers maneuvering off the petroleum transshipment facilities on St. Eustatius, potential petroleum spillage and subsequent use of dispersants, general vessel passage in a zone of high maritime traffic, possible overfishing for certain species, and exploration for petroleum reserves (so far unsuccessful). Saba Bank's fisheries and dive operations are economically significant to the small community on Saba Island (about 1500 residents) that has direct responsibility for its management.

The known fish fauna of Saba Bank prior to our survey consisted of 42 fish species. Most of these species were taken during fishery bottom-trawl surveys on Saba Bank, including two M/V Oregon stations in 1958 and nine stations in 1959, and two trawl hauls taken in 1969 by the R/V Pillsbury. Although four of these trawls were taken on or near the top of Saba Bank, nine were on the deep outer slopes. The habitats sampled during these surveys were restricted to relatively soft-bottom habitats due to the exclusive use of trawling techniques. These trawl samples provided valuable records of fishes living on soft bottoms and on the outer slopes of Saba Bank.

A biodiversity-assessment survey was carried out on Saba Bank during 2006 and 2007, with a major goal being to improve knowledge of the biodiversity on one of the world's most significant, though poorly known, coral-capped seamounts. In an effort to record as many fish species as possible in the short period of time available for the survey, we utilized a variety of fish sampling techniques. These techniques included visual surveys by divers, use of SCUBA to apply ichthyocide (a natural fish toxicant consisting of dried and powdered *Derris* root - assayed at 7.5% rotenone), hand-line fishing, by-catch from lobster and fish traps taken by local fishermen, and port sampling observations of fish landings. During the surveys, an attempt was made to obtain a photograph documenting the fresh colors of as many species as possible, a tissue sample of each species, and preserved specimen vouchers that have been archived in the National fish collection (USNM) of the National Museum of Natural History (NMNH), Smithsonian Institution (SI). Specimens representing six or more undescribed species and two rare gobies, *Pycnomma roosevelti* and *Psilotris boehlkei*, were collected.

The habitats surveyed on the Saba Bank “atoll” during this study are classified as: fore reef, patch reef, lagoonal and Small Bank. Fore-reef areas are located around the outer rim of the submerged atoll. Patch reef is an isolated portion of “reef” situated on the lagoonal (interior) side of the fore reef. Lagoonal is the central portion of the atoll interior to the fore-reefs around the atoll rim and may have a variety of bottom types. Small Bank is a small, independent “seamount-like” structure located off the northwest corner of Saba Bank with its shallowest depth at about 11 m. Depth categories were arbitrarily assigned as shallow (11–24 m), mid-depth (25–34 m), and deep (35–38 m).

The primary goals of the overall biodiversity survey were to provide data and analysis to support designation of Saba Bank as a marine protected area, to support the development of a management plan, and to contribute to a petition to the International Maritime Organization to designate appropriate parts of Saba Bank as a Particularly Sensitive Sea Area.

## Results

**Figure 1:**
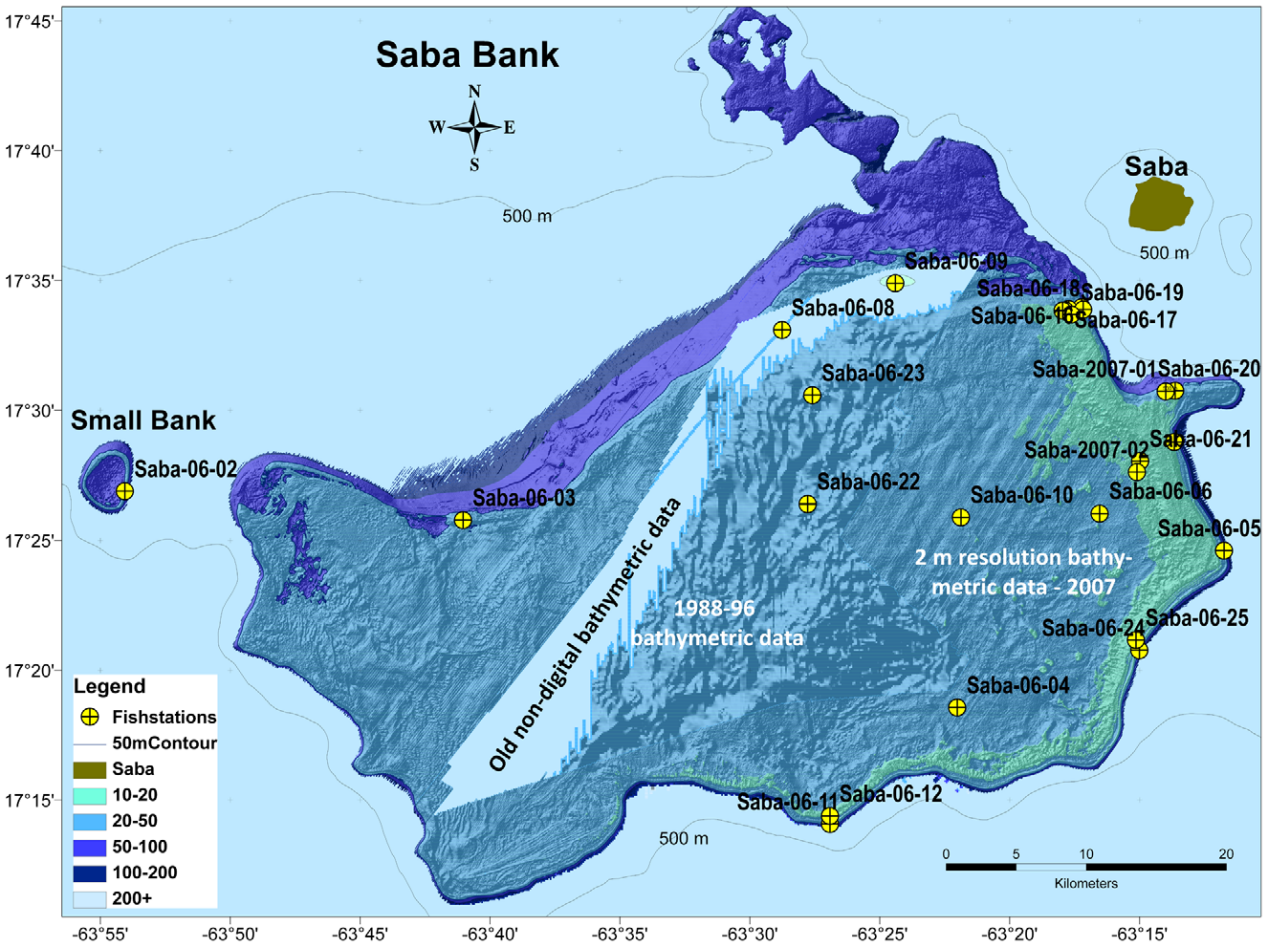
Bathymetric map of Saba Bank with fish stations marked.

We occupied fish stations at 25 locations during a rapid assessment (RAP) survey, 2–16 January 2006; including: 20 ichthyocide stations, 12 roving visual surveys, two hook & line stations, and five by-catch stations from lobster traps; two fish-ichthyocide stations were occupied at two additional locations on 20 June 2007 (Figure 1). In 2007, Toller assessed the benthic communities and fish assemblages based in part on 40 visual surveys of the fish fauna at an area on the eastern side of Saba Bank.

Specimens collected during the RAP survey were preserved as vouchers and processed into the fish collection (available online at http://vertebrates.si.edu/fishes/fishes_collections.html) at the NMNH (as many species as possible were also tissue sampled and photographed in the field). Toller collected and preserved vouchers from fishery landings when possible and photographed specimens representing new records. Toller's vouchers were processed into the fish collection at the NMNH and are also available in the online database.

Some species were only taken during fishery bottom trawl surveys by the M/V Oregon (1958, 1959) and the R/V Pillsbury (1969) on Saba Bank. Species represented by voucher specimens in museum collections are included in our comprehensive species list.

**Figure 2:**
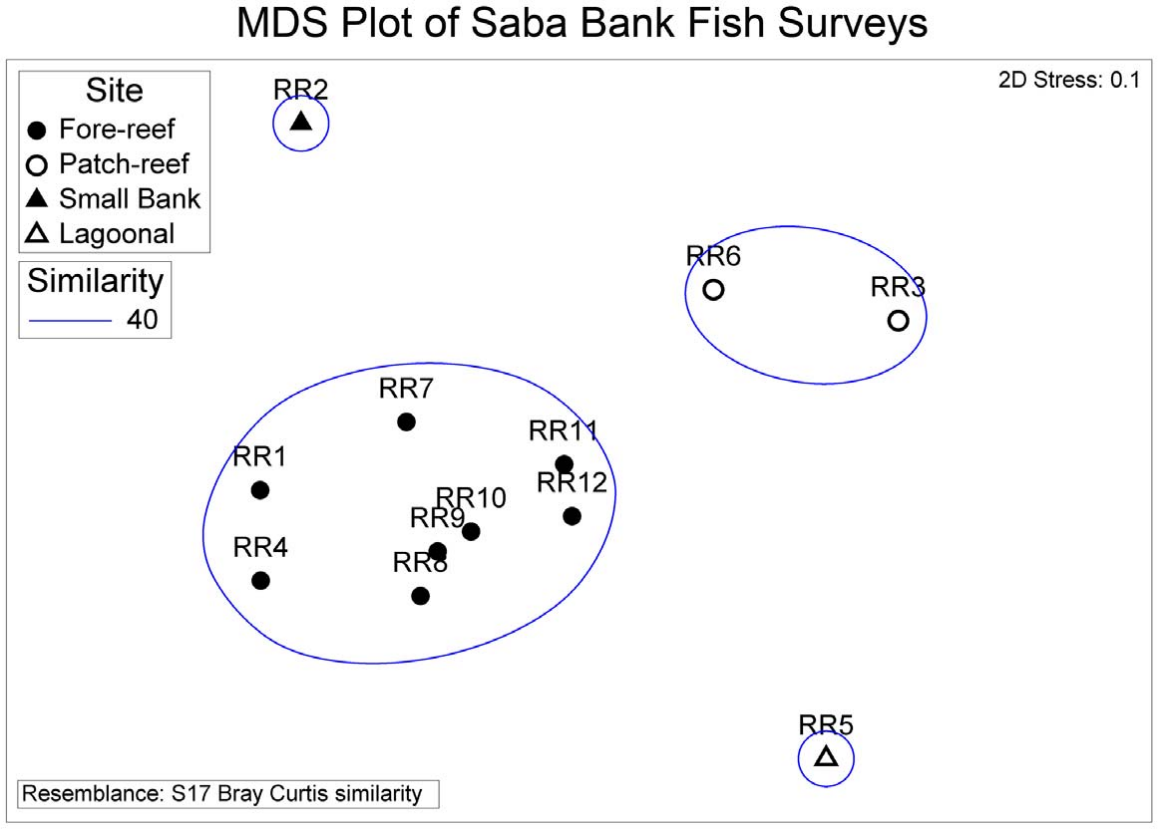
MDS ordination of fish-survey stations illustrates high similarity of assemblages within fore-reef sites and significant differences (ANOSIM, *P*<0.05) among habitats.

A non-metric multidimensional-scaling (MDS) ordination of the 12 rotenone and roving stations based on a Bray-Curtis similarity matrix of incidence data was used to illustrate similarities and differences of fish assemblages found on the four atoll habitat types: fore reef, patch reef, lagoonal, Small Bank (Figure 2). There were significant differences among habitat types (ANOSIM Global R = 0.96, *P* = 0.001). Differences between fore-reef stations and patch-reef stations were most pronounced (ANOSIM, Pairwise R = 0.92, *P* = 0.002). Fore-reef assemblages ranged from 39 to 60 species per station while the patch-reef stations had 26 and 32 species. Fore-reef stations were 50% similar to each other, and patch-reef stations were up to 60% similar. Fore-reef assemblages were not significantly different than Small Bank, or the lagoonal habitat (ANOSIM, Pairwise R = 0.973, P = 0.11). Low sample size (one station with 39 species) in the lagoonal habitat limits this comparison.

There were significant differences in the fish assemblages when classified by depth - shallow, middle, and deep (ANOSIM Global test, R = 0.618, P = 0.006). Differences in species composition were most evident between mid-depth and deep sites (ANOSIM Pairwise test, R = 0.829, P = 0.008). Fore-reef sites were typically at mid to shallow depths (20 to 34 m) whereas the patch-reef sites were typically deep (35 to 38 m). There is some evidence of habitat heterogeneity and vertical zonation for fish assemblages on Saba Bank, but more sampling is necessary to discern whether habitat or depth best explains the differences among groups.

The horizontal axis in the MDS plot represents a gradient in species richness with patch-reef and lagoonal sites having lowest richness ( Figure 2). The vertical axis in the MDS plot illustrates differences related to depth with deepest station (Small Bank) higher in the vertical axis, and shallower sites lower in the vertical axis. The two-dimensional stress value was low in the MDS (stress = 0.1) indicating a slight chance of misrepresentation.

A principal components analysis (PCA) was used to understand which species assemblages were responsible for differences among stations. The first three components explained 43.9% of the variation. The first component (18.6% of variation explained) was dominated by ubiquitous species and most common species. Strongest loadings (negative) on the first principal component (PC1) included the four species found at all stations (*Halichoeres garnoti*, *Serranus tigrinus*, *Stegastes partitus*, and *Thalassoma bifasciatum*) and 13 species found at all but one to four of the stations. These 13 common species are rarely found at the small bank, patch-reef, and lagoonal stations and, therefore, PC1 also serves to define the fore-reef sites. For example, *Acanthurus bahianus*, *Coryphopterus glaucofraenum*, *Coryphopterus dicrus*, and *Scarus taeniopterus* were found at all fore-reef sites but rarely at the Small Bank, lagoonal and at least one of the patch-reef sites. The strongest positive loadings on PC1 are from 11 species only found at the lagoonal site. Principal components 2 and 3 correspond strongly to the horizontal or species richness component on the MDS with strongest positive loadings on species typically found at the low to medium species richness stations 3, 5, 6 and 8–12 (e.g. *Cryptotomus roseus, Haemulon melanurum, Halichoeres bivittatus, Astrapogon puncticulatus*, and *Serranus baldwini*). Strongest negative scores were on species that were frequently found at high to medium species richness stations 1, 2, 4, 7 and 8 (e.g. *Hypoplectrus puella, Lythrypnus elasson, Prognathodes aculeatus, Neoniphon marianus*, and *Gramma loreto*).

An annotated list of the fishes of Saba Bank is provided below. In the list, we include the family, genus and species, author and English common name (as common names are not standardized internationally, we strove to apply the most widely used English common name based on FishBase (http://www.fishbase.org) listings). The use of “cf” before a species name indicates that the specimen photographed is similar to that species, but probably represents an undescribed species. Voucher specimens are archived at the National Museum of Natural History (USNM) and the Florida Museum of Natural History (UF) and each species with vouchers is annotated with the museum's acronym where the specimens are housed. The basis of each species record is indicated by: I – ichthyocide station, F – caught by a local fisherman and photographed, T – bottom trawl, O – visual sighting during Toller survey, V – visual sighting during RAP survey at roving and rotenone station. Lengths of specimens are recorded in mm for either standard length (SL), total length (TL), or fork length (FL). Photographs showing the color pattern of freshly collected specimens are included for as many of the species as possible. Images illustrating observed sexual and developmental (juvenile to adult) variability in color pattern are included where possible.

### Ginglymostomatidae—nurse sharks

**Figure 3:**
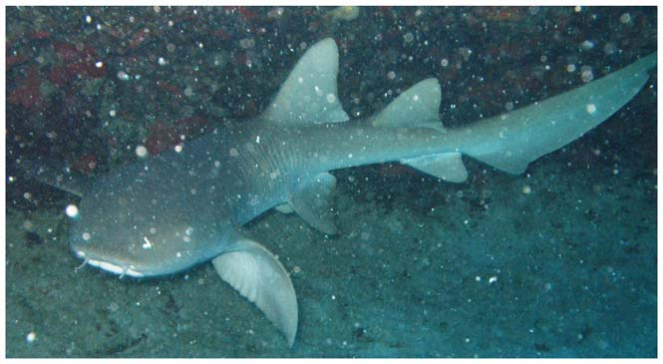
*Ginglymostoma cirratum*, underwater photo by Juan Sanchez.

*Ginglymostoma cirratum* (Bonnaterre, 1788)*—*nurse shark; **OV**; Figure 3

### Squalidae—dogfish sharks

**Figure 4:**
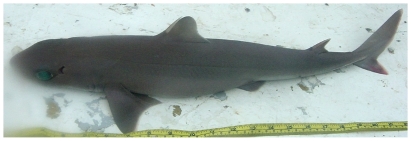
*Squalus cubensis*, 475 mm TL, photo by W Toller.

*Squalus cubensis* Howell Rivero, 1936*—*Cuban dogfish; **F**; Figure 4

### Carcharhinidae—requiem sharks

*Carcharhinus perezii* (Poey, 1876)*—*reef shark; **F**

*Galeocerdo cuvier* (Péron & Lesueur, 1822)*—*tiger shark; **F,O**

### Etmopteridae—lantern sharks

*Etmopterus bullisi* Bigelow & Schroeder, 1957*—*lined lantern shark; **USNM, T**

### Dasyatidae—whiptail stingrays

*Dasyatis americana* Hildebrand & Schroeder, 1928*—*southern stingray; **V**

### Muraenidae—morays

*Anarchias similis* (Lea, 1913)*—*pygmy moray; **USNM, I**

**Figure 5:**
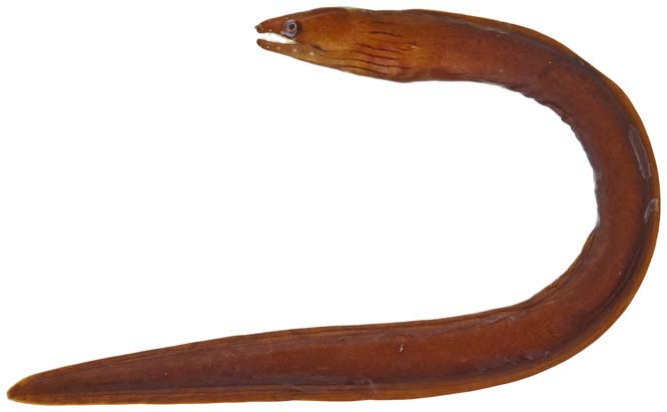
*Enchelychore carychroa*, 175 mm TL, photo by JT Williams.

*Enchelycore carychroa* (Böhlke & Böhlke, 1976)*—*chestnut moray; **USNM, I, F;** Figure 5

*Enchelycore nigricans* (Bonnaterre, 1788)*—*viper moray; **USNM, I**

**Figure 6:**
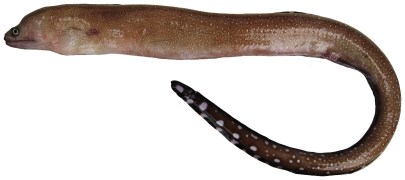
*Gymnothorax conspersus*, 730 mm TL, photo by W Toller.

*Gymnothorax conspersus* Poey, 1867*—*saddled moray; **USNM, F**; Figure 6

**Figure 7:**
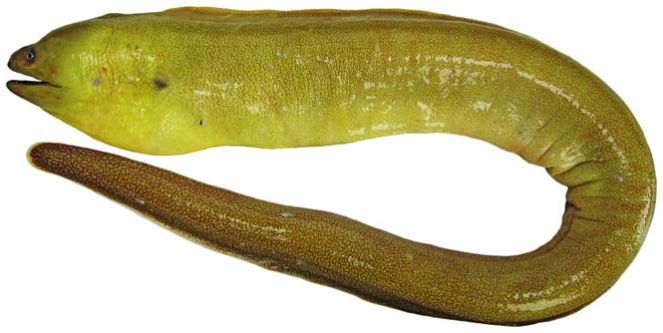
*Gymnothorax maderensis*, 300 mm TL, photo by W Toller.

*Gymnothorax maderensis*(Johnson, 1862)*—*sharktooth moray; **USNM, F**; Figure 7

**Figure 8:**
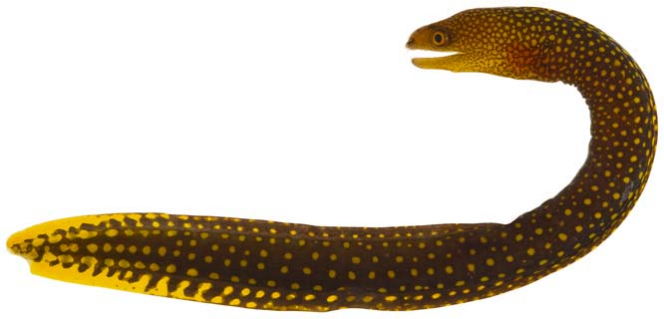
*Gymnothorax miliaris*, 60.3 mm TL, photo by JT Williams.

*Gymnothorax miliaris* (Kaup, 1856)*—*goldentail moray; **USNM, I, O**; Figure 8

**Figure 9:**
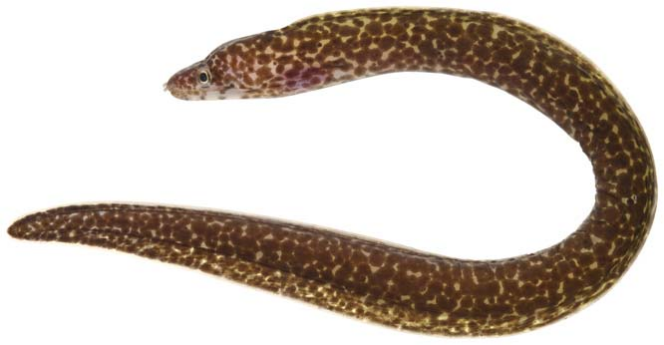
*Gymnothorax moringa*, 189.8 mm TL, photo by JT Williams.

*Gymnothorax moringa* (Cuvier, 1829)*—*spotted moray; **USNM, I, O, V**; Figure 9

**Figure 10:**
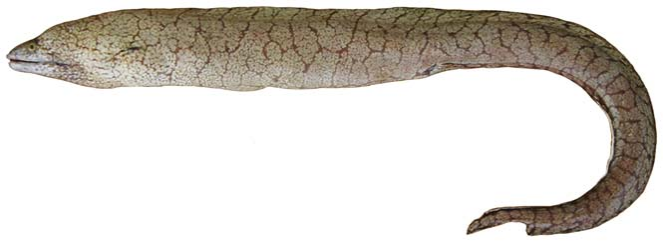
*Gymnothorax polygonius*, 810 mm TL, photo by W Toller.

*Gymnothorax polygonius* Poey, 1876*—*polygon moray; **USNM, F**; Figure 10

**Figure 11:**
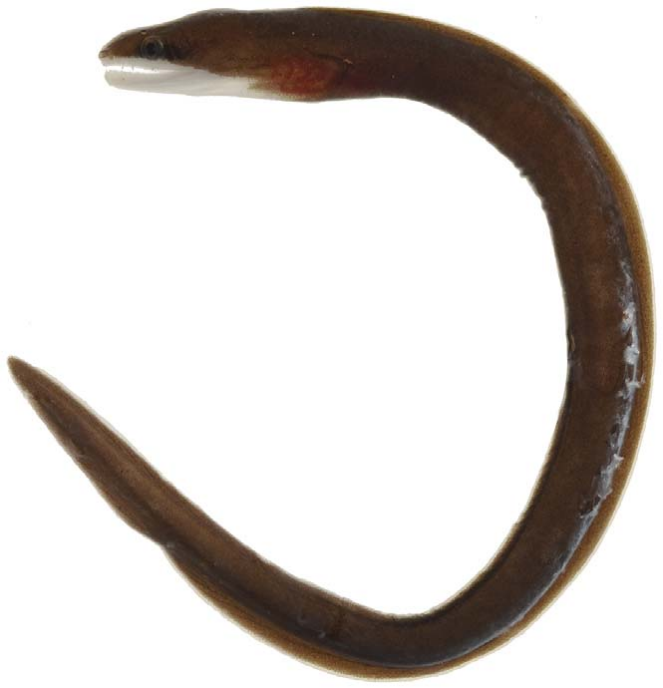
*Gymnothorax vicinus*, 48.2 mm TL, photo by JT Williams.

*Gymnothorax vicinus* (Castelnau, 1855)*—*purplemouth moray; **USNM, I, O**; Figure 11

**Figure 12:**
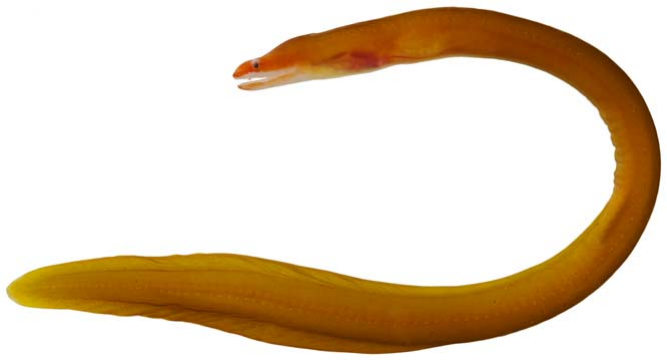
*Monopenchelys acuta*, 132.4 mm TL, photo by JT Williams.

*Monopenchelys acuta* (Parr, 1930)*—*redface moray; **USNM, I**; Figure 12

*Uropterygius macularius* (Lesueur, 1825)*—*marbled moray; **USNM, I**

### Ophichthidae—snake eels

**Figure 13:**
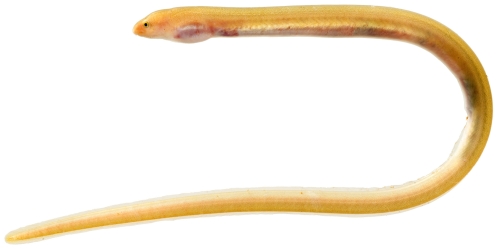
*Ahlia egmontis*, 183.0 mm TL, photo by JT Williams.

**Figure 14:**
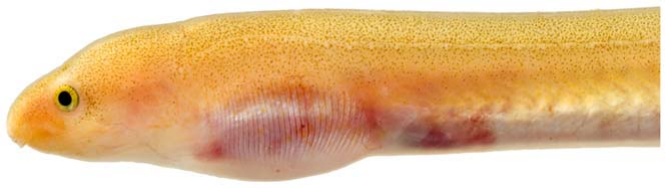
*Ahlia egmontis*, 183.0 mm TL, close-up of head, photo by JT Williams.

*Ahlia egmontis* (Jordan, 1884)*—*key worm eel; **USNM, I**; Figures 13, 14

**Figure 15:**
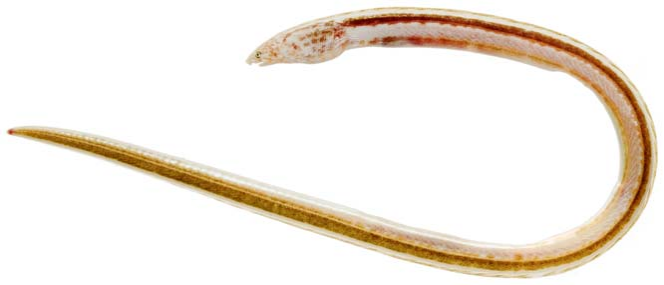
*Aprognathodon platyventris*, 149.2 mm TL, photo by JT Williams.

**Figure 16:**
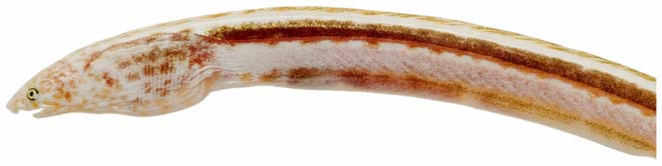
*Aprognathodon platyventris*, 149.2 mm TL, close-up of head, photo by JT Williams.

*Aprognathodon platyventris* Böhlke, 1967*—*stripe eel; **USNM, I**; Figures 15, 16

*Myrichthys breviceps* (Richardson, 1848) *—*sharptail eel; **O**

**Figure 17:**
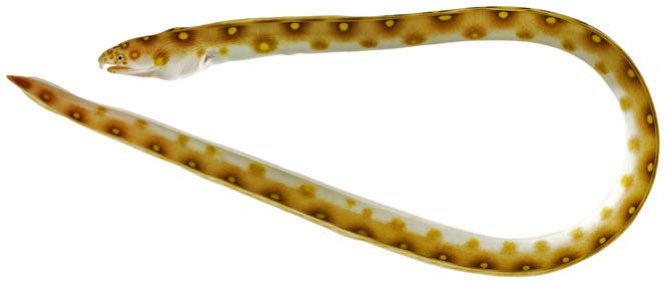
*Myrichthys ocellatus*, 383 mm TL, photo by JT Williams.

**Figure 18:**
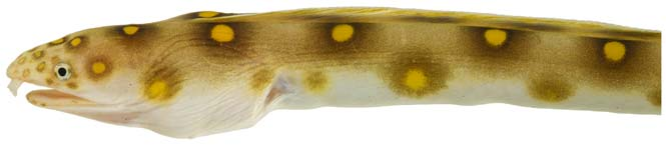
*Myrichthys ocellatus*, 383 mm TL, close-up of head, photo by JT Williams.

*Myrichthys ocellatus* (Lesueur, 1825)*—*goldspotted eel; **USNM, I**; Figures 17, 18

### Congridae—conger eels

*Bathycongrus thysanochilus* (Reid, 1934)*—*conger eel; **USNM, T**

*Conger esculentus* Poey, 1866*—*grey conger; **USNM, F**

### Chlopsidae—false morays

**Figure 19:**
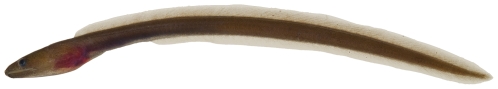
*Kaupichthys hyoproroides*, 79.4 mm TL, photo by JT Williams.

*Kaupichthys hyoproroides* (Strömman, 1896)*—*false moray; **USNM, I**; Figure 19

**Figure 20:**
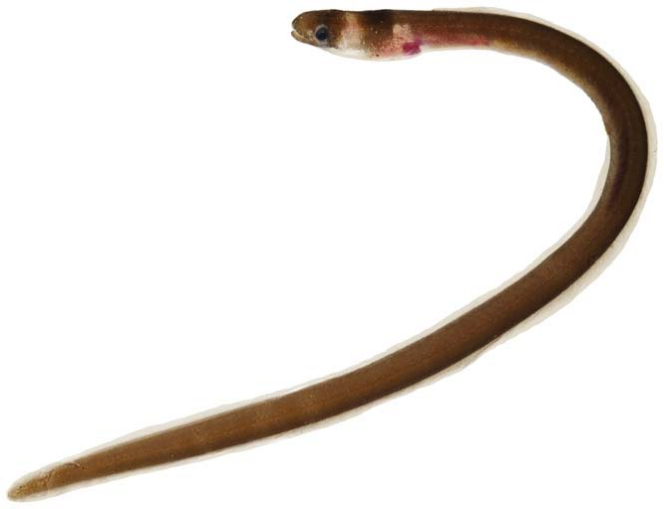
*Kaupichthys nuchalisi*, 61.6 mm TL, photo by JT Williams.

*Kaupichthys nuchalis* Böhlke, 1967*—*collared eel; **USNM, I**; Figure 20

### Moringuidae—spaghetti eels

**Figure 21:**
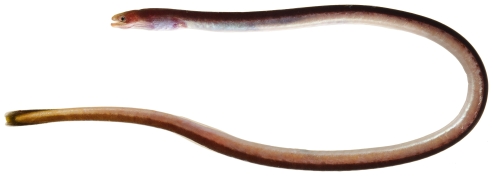
*Moringua edwardsi*, 258 mm TL, photo by JT Williams.

*Moringua edwardsi* (Jordan & Bollman, 1889)*—*spaghetti eel; **USNM, I**; Figure 21

### Synodontidae—lizardfishes

*Saurida brasiliensis* Norman 1935*—*largescale lizardfish; **UF, T**

*Saurida normani* Longley 1935*—*shortjaw lizardfish; **UF, T**

**Figure 22:**
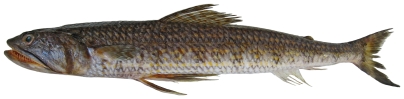
*Synodus intermedius*, 316 mm SL, photo by W Toller.

*Synodus intermedius* (Spix & Agassiz, 1829)*—*sand diver; **USNM, UF, F, O**; Figure 22

*Synodus poeyi* Jordan, 1887*—*offshore lizardfish; **UF, T**

*Synodus saurus* (Linnaeus, 1758)*—*Atlantic lizardfish; **UF, I, V**

**Figure 23:**
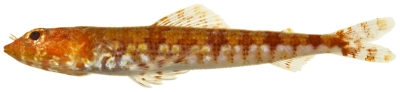
*Synodus synodus*, 42.5 mm SL, photo by JT Williams.

*Synodus synodus* (Linnaeus, 1758)*—*red lizardfish; **UF, USNM, I;** Figure 23

*Trachinocephalus myops* (Forster, 1801)*—*snakefish; **V**

### Ophidiidae—cusk-eels

**Figure 24:**
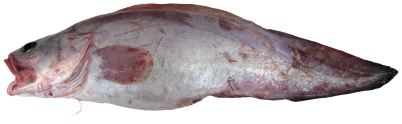
*Brotula barbata*, 628 mm TL, photo by W Toller.

*Brotula barbata* (Bloch & Schneider, 1801)*—*bearded brotula; **USNM, O**; Figure 24

*Neobythites ocellatus* Günther 1887*—*ocellate cusk-eel; **USNM, T**

*Neobythites unicolor* Nielsen & Retzer 1994*—*unicolor cusk-eel; **USNM, T**

*Ophidion antipholus* Lea & Robins, 2003*—*longnose cusk-eel; **UF, T**

*Otophidium omostigma* (Jordan & Gilbert, 1882)*—*polka-dot cusk-eel; **UF, T**

**Figure 25:**
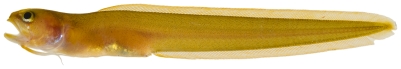
*Parophidion schmidti*, 69.3 mm TL, photo by JT Williams.

*Parophidion schmidti* (Woods & Kanazawa, 1951)*—*dusky cusk-eel; **USNM, I**; Figure 25

**Figure 26:**
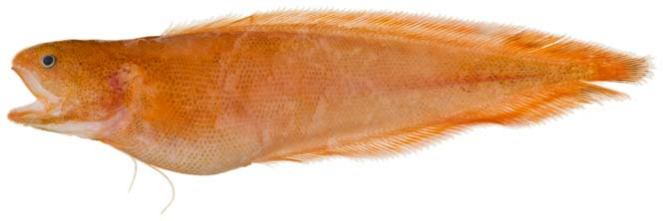
*Petrotyx sanguineus*, 62.2 mm TL, photo by JT Williams.

*Petrotyx sanguineus* (Meek & Hildebrand, 1928)*—*redfin brotula; **USNM, I**; Figure 26

### Bythitidae—viviparous brotulas

**Figure 27:**
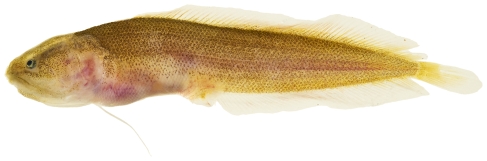
*Ogilbia sabaji*, 27.2 mm SL, photo by JT Williams.

*Ogilbia sabaji* Moller, Schwarzhans & Nielsen, 2005*—*Sabaj coralbrotula; **USNM, I**; Figure 27

### Antennariidae—frogfishes

**Figure 28:**
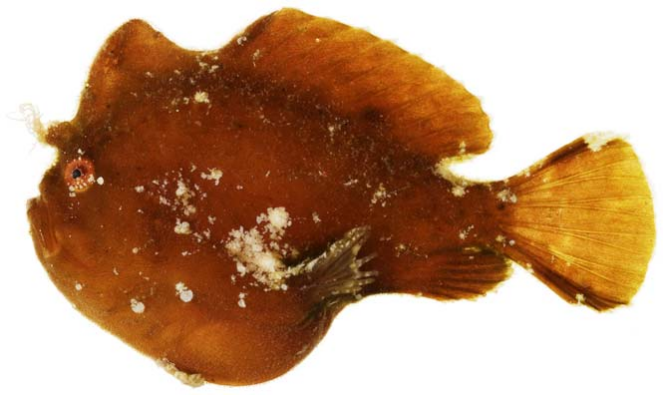
*Antennarius pauciradiatus*, 24.9 mm SL, photo by JT Williams.

**Figure 29:**
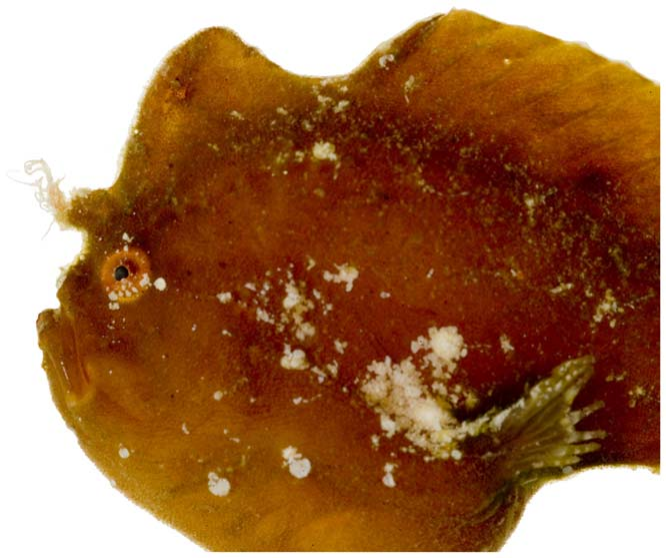
*Antennarius pauciradiatus*, 24.9 mm SL, close-up of head, photo by JT Williams.

*Antennarius pauciradiatus* Schultz, 1957 *—*dwarf frogfish; **USNM, I**; Figures 28, 29

*Antennarius multiocellatus* (Valenciennes, 1837) *—*longlure frogfish; **USNM, I**

A single juvenile specimen was collected in 2007. Although adults of this species have a very long first spine, our juvenile specimen has the first dorsal spine about the same length as the second. Böhlke and Chaplin [3] mention this allometric growth pattern in which the first spine is short in young specimens, but increases in length with growth. The juvenile exhibits the typical adult color pattern.

### Chaunacidae—sea toads

*Chaunax suttkusi* Caruso, 1989*—*Suttkus sea toad; **USNM, T**

### Ogcocephalidae—batfishes

All of the batfish records from Saba Bank are based on trawl collections with vouchered museum specimens.

*Dibranchus atlanticus* Peters, 1876*—*Atlantic batfish; **UF, T**

*Halieutichthys aculeatus* (Mitchill, 1818)*—*pancake batfish; **UF, T**

*Ogcocephalus pumilus* Bradbury, 1980*—*dwarf batfish; **USNM, T**

### Exocoetidae—flyingfishes

*Cypselurus comatus* (Mitchill, 1815)*—*clearwing flyingfish; **USNM**

This specimen was probably captured at the surface using a dip net.

### Syngnathidae—pipefishes

*Anarchopterus tectus* (Dawson, 1978) *—*insular pipefish; **USNM, I**

**Figure 30:**
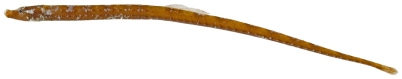
*Bryx randalli*, 24.3 mm SL, photo by JT Williams.

*Bryx randalli* (Herald, 1965)*—*ocellated pipefish; **USNM, I**; Figure 30

**Figure 31:**
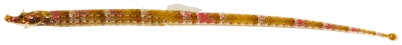
*Micrognathus crinitus*, 102.8 mm SL, photo by JT Williams.

**Figure 32:**
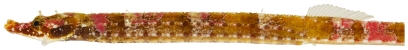
*Micrognathus crinitus*, 102.8 mm SL, close-up of head, photo by JT Williams.

*Micrognathus crinitus* (Jenyns, 1842) *—*banded pipefish; **USNM, I**; Figures 31, 32

### Aulostomidae—trumpetfishes

*Aulostomus maculatus* Valenciennes, 1837*—*trumpetfish; **USNM, I**, **O**, **V**

### Holocentridae—squirrelfishes

**Figure 33:**
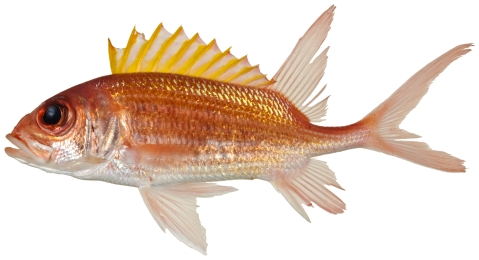
*Holocentrus ascensionus*, 195.0 mm SL, photo by JT Williams.

*Holocentrus adscensionis* (Osbeck, 1765)*—*squirrelfish; **USNM, I, O, V**; Figure 33

**Figure 34:**
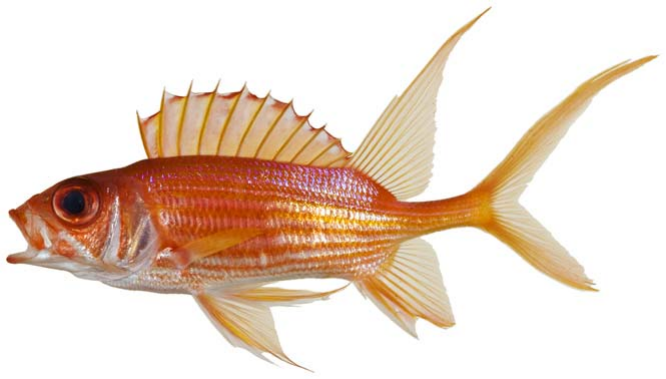
*Holocentrus rufus*, 153.2 mm SL, photo by JT Williams.

*Holocentrus rufus* (Walbaum, 1792)*—*longspine squirrelfish; **USNM, I, O, V**; Figure 34

**Figure 35:**
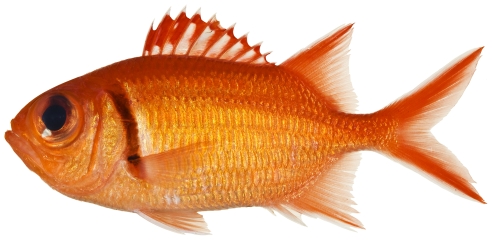
*Myripristis jacobus*, 114.7 mm SL, photo by JT Williams.

*Myripristis jacobus* Cuvier, 1829*—*blackbar soldierfish; **USNM, I, O**; Figure 35

**Figure 36:**
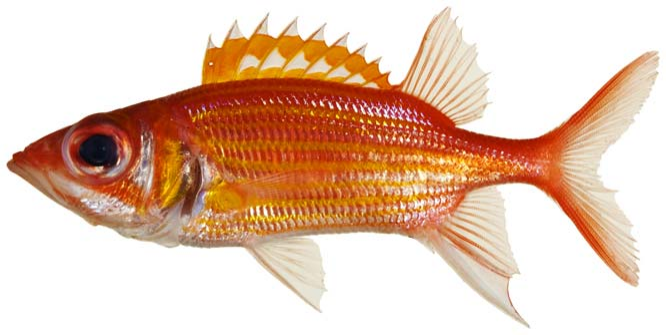
*Neoniphon marianus*, 98.2 mm SL, photo by JT Williams.

*Neoniphon marianus* (Cuvier, 1829)*—*longjaw squirrelfish; **USNM, I**; Figure 36

**Figure 37:**
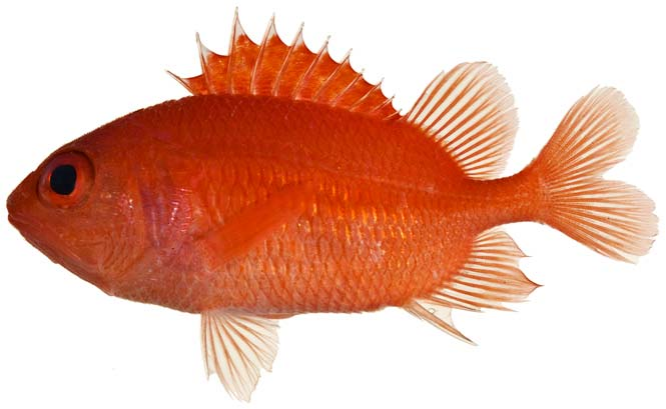
*Plectrypops retrospinis*, 71.5 mm SL, photo by JT Williams.

*Plectrypops retrospinis* (Guichenot, 1853)*—*cardinal soldierfish; **USNM, I**; Figure 37

**Figure 38:**
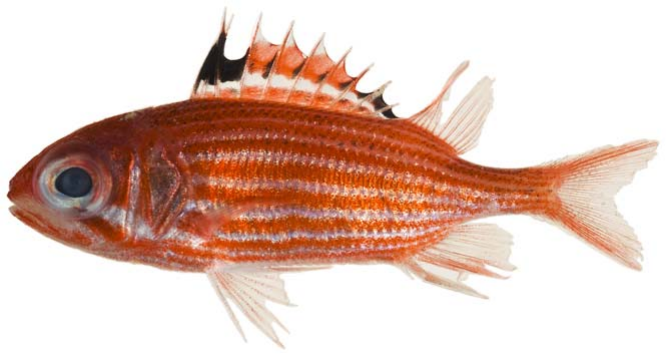
*Sargocentron coruscum*, juvenile, 31.1 mm SL, photo by JT Williams.

**Figure 39:**
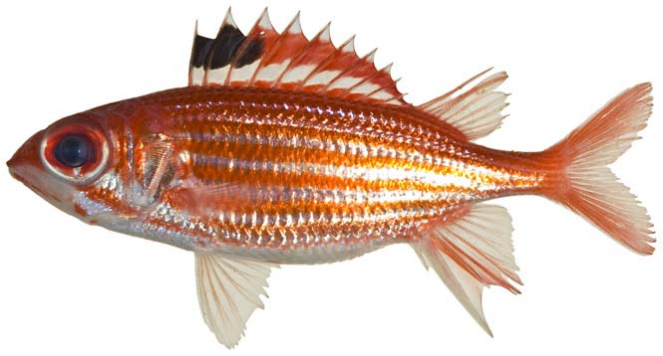
*Sargocentron coruscum*, 81.7 mm SL, photo by JT Williams.

*Sargocentron coruscum* (Poey, 1860)*—*reef squirrelfish; **USNM, I, O**; Figures 38, 39

### Scorpaenidae—scorpionfishes

**Figure 40:**
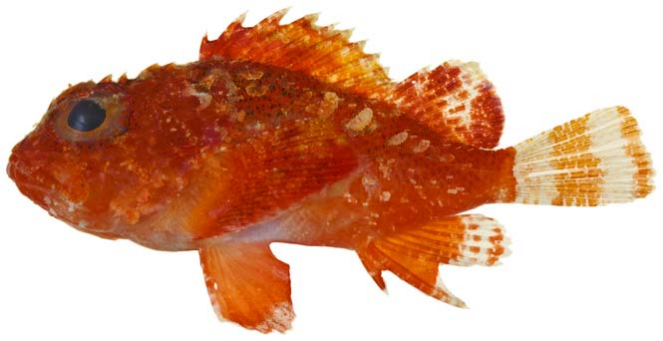
*Scorpaena albifimbria*, 37.4 mm SL, photo by JT Williams.

*Scorpaena albifimbria* Evermann & Marsh, 1900*—*coral scorpionfish; **USNM, I**; Figure 40

*Scorpaena bergii* Evermann & Marsh, 1900*—*goosehead scorpionfish; **USNM, I**

**Figure 41:**
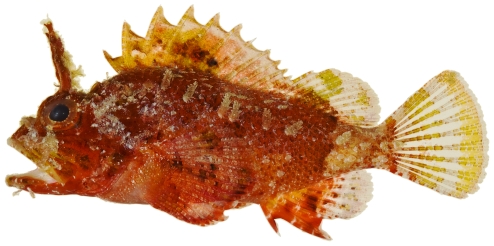
*Scorpaena grandicornis*, 65.1 mm SL, photo by JT Williams.

*Scorpaena grandicornis* Cuvier, 1829*—*plumed scorpionfish; **USNM, I**; Figure 41

**Figure 42:**
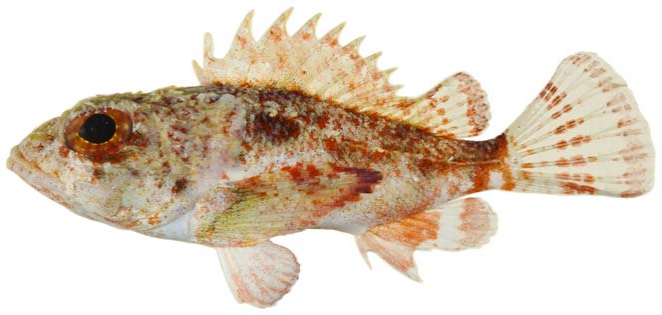
*Scorpaena inermis*, 40.1 mm SL, red morph, photo by JT Williams.

**Figure 43:**
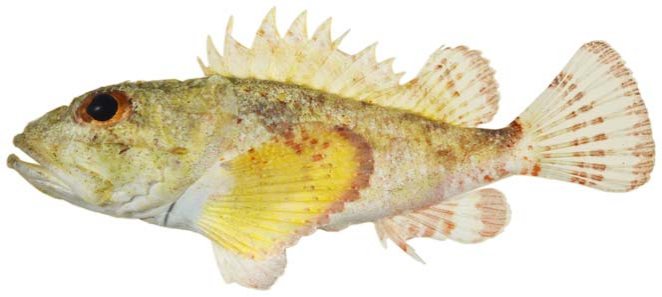
*Scorpaena inermis*, 65.9 mm SL, yellow morph, photo by JT Williams.

*Scorpaena inermis* Cuvier, 1829 *—*mushroom scorpionfish; **USNM, I, T**; Figures 42, 43

Two color morphs were collected at Saba Bank, a red morph (Figure 42) and a yellow morph (Figure 43).

*Scorpaena plumieri* Bloch, 1789*—*spotted scorpionfish; **USNM, I**

**Figure 44:**
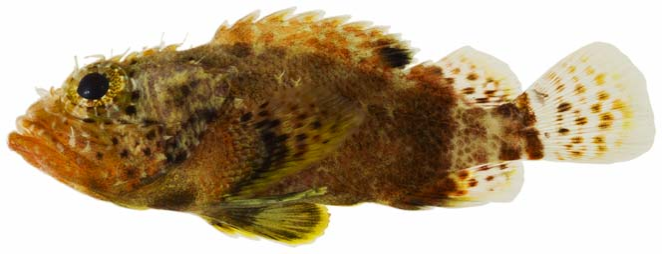
*Scorpaenodes caribbaeus*, 30.1 mm SL, photo by JT Williams.

*Scorpaenodes caribbaeus* Meek & Hildebrand, 1928*—*reef scorpionfish; **USNM, I**; Figure 44

### Triglidae—searobins

All of the searobin records from Saba Bank are based on trawl collections with vouchered museum specimens.

*Bellator egretta* (Goode & Bean, 1896)*—*streamer searobin; **USNM, T**

*Bellator militaris* (Goode & Bean, 1896)*—*horned searobin; **UF, T**

*Prionotus ophryas* Jordan & Swain, 1885*—*bandtail searobin; **UF, T**

### Symphysanodontidae—slopefishes

**Figure 45:**
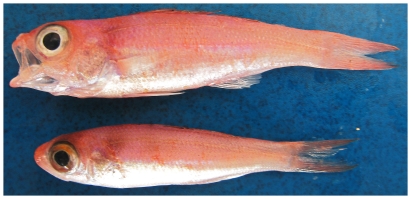
*Symphysanodon berryi*, top 75 mm SL, lower 63 mm SL, photo by W Toller.

*Symphysanodon berryi* Anderson, 1970*—*slope bass; **USNM, F**; Figure 45

### Serranidae—sea basses

**Figure 46:**
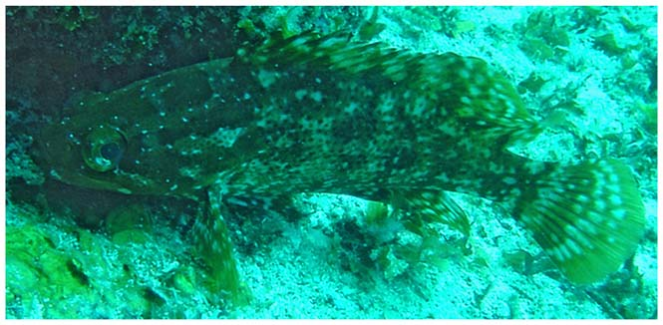
*Alphestes afer*, underwater photo by W Toller.

*Alphestes afer* (Bloch, 1793)*—*mutton hamlet; **F, O**; Figure 46

**Figure 47:**
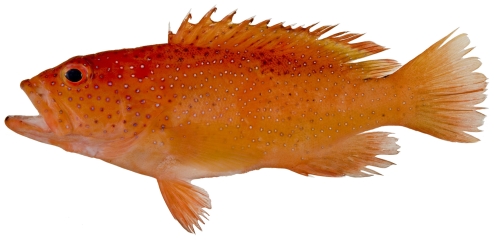
*Cephalopholis cruentata*, 101.1 mm SL, photo by JT Williams.

*Cephalopholis cruentata* (Lacepède, 1802)*—*graysby; **USNM, I, F, O, V**; Figure 47

**Figure 48:**
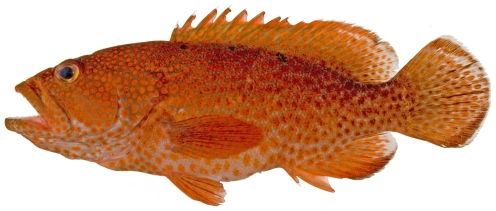
*Cephalopholis fulva*, 121.1 mm SL, photo by JT Williams.

*Cephalopholis fulva* (Linnaeus, 1758)*—*coney; **UF,** **USNM, I, F, O, V**; Figure 48

*Diplectrum bivittatum* (Valenciennes, 1828) *—*dwarf sand perch; **UF, T**

**Figure 49:**
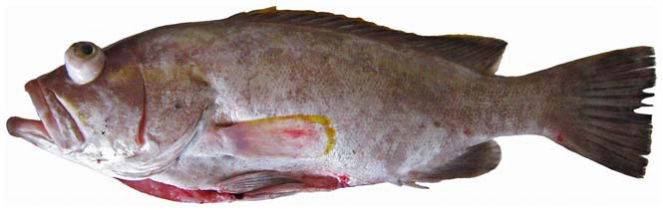
*Epinephelus flavolimbatus*, 652 mm SL, photo by W Toller.

*Epinephelus flavolimbatus* Poey, 1865*—*yellowedge grouper; **F**; Figure 49

**Figure 50:**
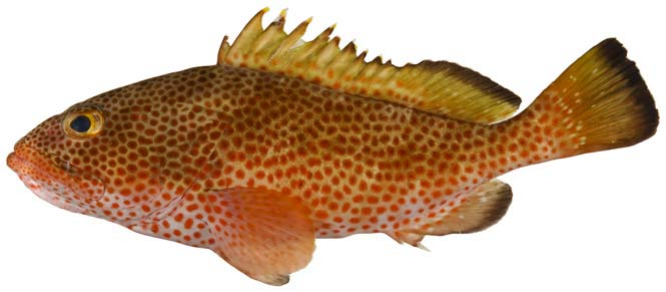
*Epinephelus guttatus*, 283.2 mm SL, photo by JT Williams.

*Epinephelus guttatus* (Linnaeus, 1758)*—*red hind; **UF**, **USNM, F, I, O, T, V**; Figure 50

*Epinephelus morio* (Valenciennes, 1828)*—*red grouper; **F**

**Figure 51:**
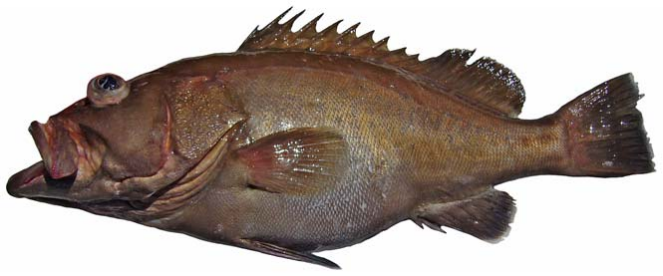
*Epinephelus niveatus*, 526 mm SL, photo by W Toller.

*Epinephelus niveatus* Valenciennes, 1828*—*snowy grouper; **USNM, F**; Figure 51

*Epinephelus striatus* (Bloch, 1792)*—*Nassau grouper; **F**

*Hypoplectrus chlorurus* (Cuvier, 1828) *—*yellowtail hamlet; **O**

**Figure 52:**
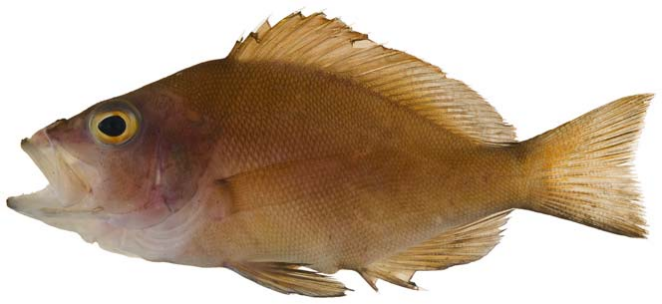
*Hypoplectrus nigricans*, 67.2 mm SL, photo by JT Williams.

*Hypoplectrus nigricans* (Poey, 1852)*—*black hamlet; **USNM, I, O, V**; Figure 52

*Hypoplectrus puella* (Cuvier, 1828)*—*barred hamlet; **O, V**

**Figure 53:**
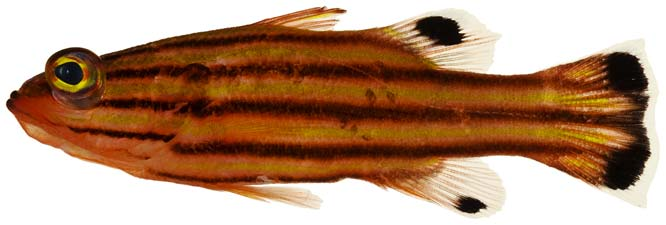
*Liopropoma rubre*, 30.0 mm SL, photo by JT Williams.

*Liopropoma rubre* Poey, 1861*—*peppermint basslet; **USNM, I**; Figure 53

**Figure 54:**
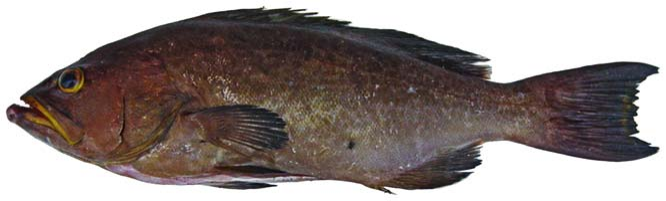
*Mycteroperca interstitialis*, 328 mm SL, photo by W Toller.

*Mycteroperca interstitialis* (Poey, 1860)*—*yellowmouth grouper; **F**; Figure 54

*Mycteroperca tigris* (Valenciennes, 1833)*—*tiger grouper; **V**

*Mycteroperca venenosa* (Linnaeus, 1758)*—*yellowfin grouper; **F, OBS**

**Figure 55:**
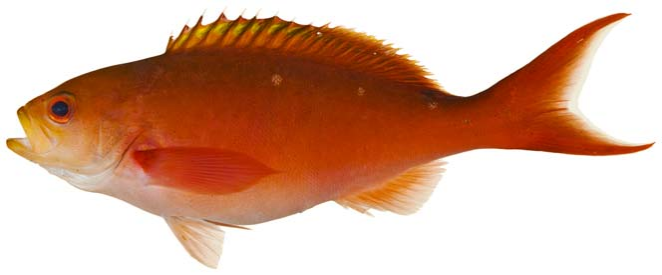
*Paranthias furcifer*, 148.4 mm SL, photo by JT Williams.

*Paranthias furcifer* (Valenciennes, 1828)*—*Atlantic creolefish; **UF**, **USNM, I, O, V**; Figure 55

**Figure 56:**
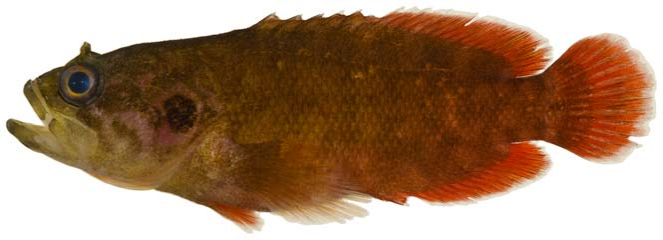
*Pseudogramma gregoryi*, 43.5 mm SL, photo by JT Williams.

*Pseudogramma gregoryi* (Breder, 1927)*—*reef bass; **USNM, I**; Figure 56

**Figure 57:**
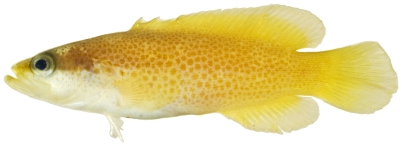
*Rypticus bistripinus*, 46.2 mm SL, photo by JT Williams.

*Rypticus bistripinus* (Mitchill, 1818) *—*freckled soapfish; **USNM, I**; Figure 57

**Figure 58:**
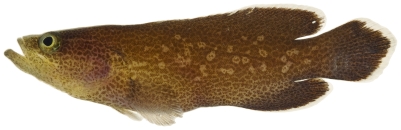
*Rypticus saponaceus*, 44.1 mm SL, photo by JT Williams.

*Rypticus saponaceus* (Bloch & Schneider, 1801)*—*greater soapfish; **USNM, I**; Figure 58

*Rypticus* new species; **USNM, I**

This new species of soapfish is very similar in appearance to *Rypticus subbifrenatus*. The new species is being described by C Baldwin and DG Smith (pers. comm.).

**Figure 59:**
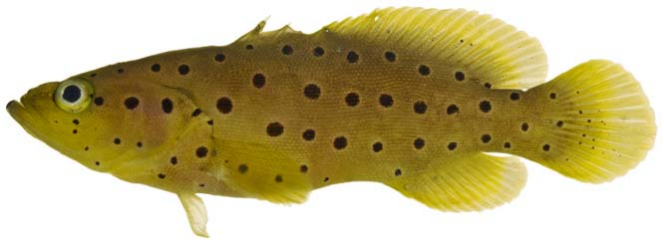
*Rypticus subbifrenatus*, 54.0 mm SL, photo by JT Williams.

*Rypticus subbifrenatus* Gill, 1861*—*spotted soapfish; **USNM, I**; Figure 59

*Schultzea beta* (Hildebrand, 1940)*—*school bass; **UF**, **USNM, I**

**Figure 60:**
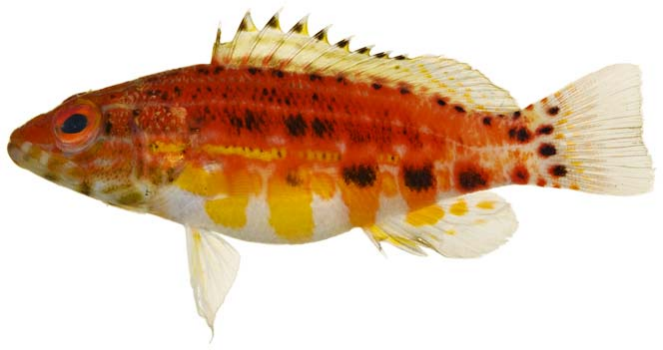
*Serranus baldwini*, 41.6 mm SL, photo by JT Williams.

*Serranus baldwini* (Evermann & Marsh, 1899)*—*lantern bass; **UF**, **USNM, I, O, V**; Figure 60

*Serranus maytagi* Robins & Starck, 1961 *—*maytag bass; **UF**

**Figure 61:**
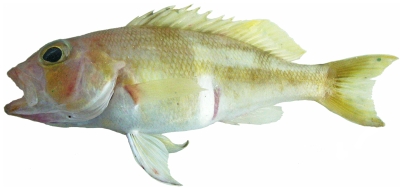
*Serranus notospilus*, 150 mm SL, photo by W Toller.

*Serranus notospilus* Longley, 1935*—*saddle bass; **USNM, F**; Figure 61

*Serranus tabacarius* (Cuvier, 1829)*—*tobaccofish; **UF, O, V**

**Figure 62:**
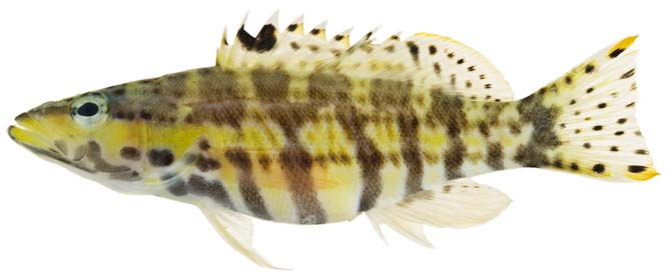
*Serranus tigrinus*, 71.1 mm SL, photo by JT Williams.

*Serranus tigrinus* (Bloch, 1790)*—*harlequin bass; **USNM, I**, **O**, **V**; Figure 62

**Figure 63:**
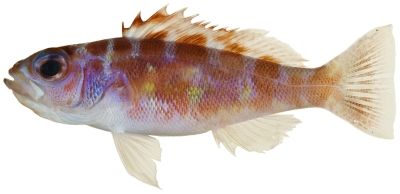
*Serranus tortugarum*, 51.8 mm SL, photo by JT Williams.

*Serranus tortugarum* Longley, 1935*—*chalk bass; **UF**, **USNM, I, O**; Figure 63

### Grammatidae—basslets

**Figure 64:**
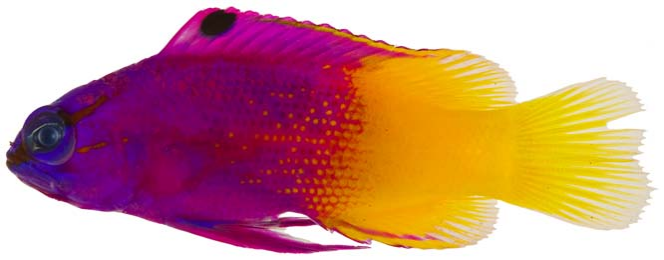
*Gramma loreto*, 38.4 mm SL, photo by JT Williams.

*Gramma loreto* Poey, 1868*—*fairy basslet; **USNM, I, O, V**; Figure 64

### Opistognathidae—jawfishes

**Figure 65:**
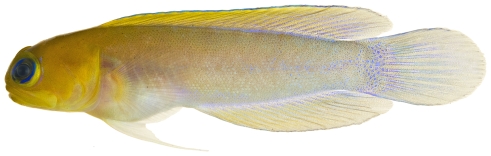
*Opistognathus aurifrons*, 44.1 mm SL, photo by JT Williams.

*Opistognathus aurifrons* (Jordan & Thompson, 1905)*—*yellowhead jawfish; **USNM, I, O, V**; Figure 65

**Figure 66:**
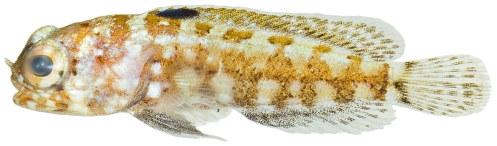
*Opistognathus whitehursti*, juvenile with large black spot in dorsal fin, 22.1 mm SL, photo by JT Williams.

**Figure 67:**
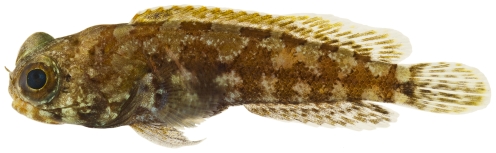
*Opistognathus whitehursti*, adult, 44.0 mm SL, photo by JT Williams.

*Opistognathus whitehursti* (Longley, 1927)*—*dusky jawfish; **USNM, I**; Figures 66, 67

### Priacanthidae—bigeyes

*Heteropriacanthus cruentatus* (Lacepède, 1801)*—*glasseye snapper; **O**

**Figure 68:**
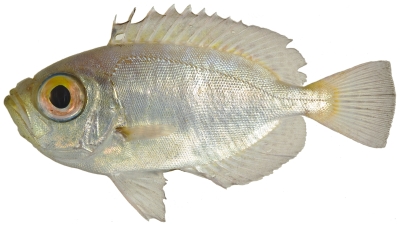
*Priacanthus arenatus*, 67.4 mm SL, photo by JT Williams.

*Priacanthus arenatus* Cuvier, 1829*—*bigeye; **USNM, I**; Figure 68

### Apogonidae—cardinalfishes

*Apogon aurolineatus* (Mowbray in Breder, 1927)*—*barred cardinalfish; **USNM, I**

*Apogon binotatus* (Poey, 1867)*—*barred cardinalfish; **USNM, I**

**Figure 69:**
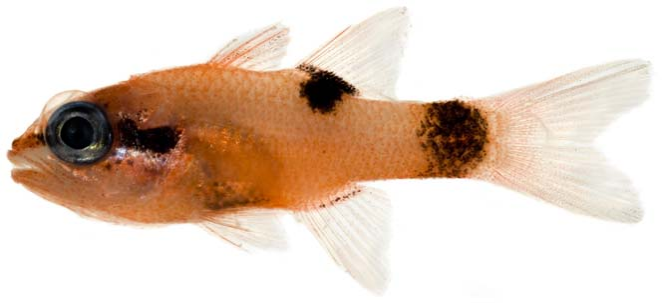
*Apogon maculatus*, 21.1 mm SL, photo by JT Williams.

*Apogon maculatus* (Poey, 1860)*—*flamefish; **USNM, I**; Figure 69

*Apogon pillionatus* Böhlke and Randall, 1968 *—*broadsaddle cardinalfish; **UF**, **USNM, I**

**Figure 70:**
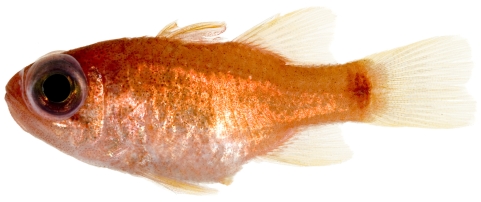
*Apogon* cf *quadrisquamatus*, 21.1 mm SL, photo by JT Williams (this specimen represents a new undescribed species.

*Apogon* cf *quadrisquamatus* Longley, 1934 *—*sawcheek cardinalfish; **USNM, I**; Figure 70

The specimens identified here as *A.*cf *quadrisquamatus* have been found to represent a new undescribed species closely related to *A. quadrisquamatus* (C. Baldwin & D.G. Smith, pers. comm. 2009).

**Figure 71:**
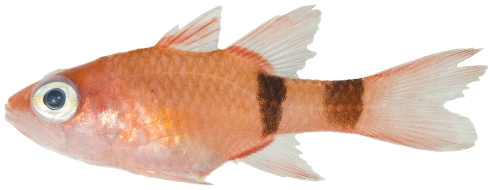
*Apogon robinsi*, 35.5 mm SL, photo by JT Williams.

*Apogon robinsi* Böhlke & Randall, 1968 *—*roughlip cardinalfish; **USNM, I**; Figure 71

**Figure 72:**
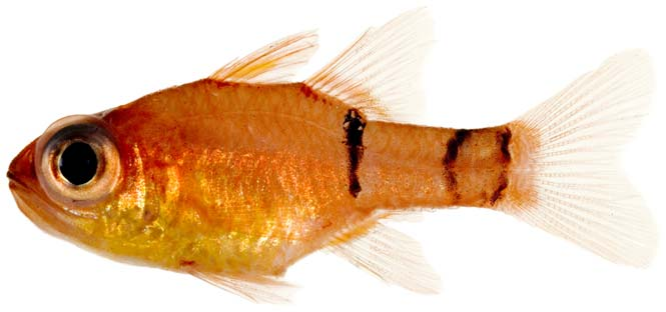
*Apogon townsendi*, 39.1 mm SL, photo by JT Williams.

*Apogon townsendi* (Breder, 1927)*—*belted cardinalfish; **USNM, I**; Figure 72

**Figure 73:**
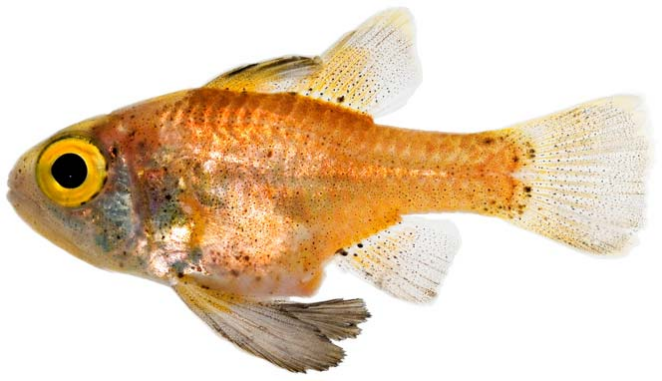
*Astrapogon puncticulatus*, 40.0 mm SL, photo by JT Williams.

*Astrapogon puncticulatus* (Poey, 1867) *—*blackfin cardinalfish; **USNM, I**; Figure 73

**Figure 74:**
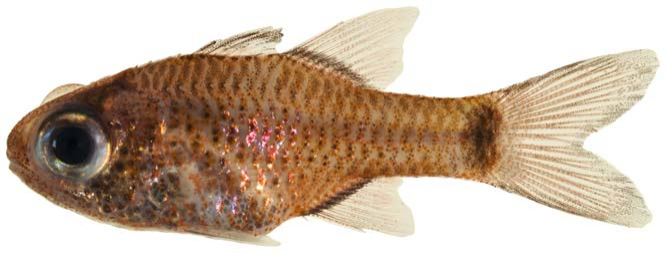
*Phaeoptyx conklini*, 40.0 mm SL, photo by JT Williams.

*Phaeoptyx conklini* (Silvester, 1916)*—*freckled cardinalfish; **USNM, I**; Figure 74

*Phaeoptyx pigmentaria* (Poey, 1860)*—*dusky cardinalfish; **USNM, I**

### Malacanthidae—tilefishes

**Figure 75:**
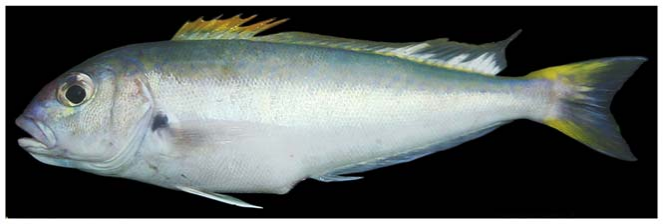
*Caulolatilus cyanops*, 288 mm SL, photo by W Toller.

*Caulolatilus cyanops* Poey, 1866*—*blackline tilefish; **USNM, F**; Figure 75

*Malacanthus plumieri* (Bloch, 1786)*—*sand tilefish; **UF, O, V**

### Coryphaenidae-dolphinfishes

*Coryphaena hippurus* Linnaeus, 1758*—*common dolphinfish; **F**

### Rachycentridae-cobias

*Rachycentron canadum* (Linnaeus, 1766)—cobia

Our record for this species is based on an underwater video recently filmed by Yap Films Inc (Toronto, Canada) at the shipwreck on Saba Bank. The video clearly shows a cobia swimming along the side of the shipwreck.

### Carangidae—jacks

**Figure 76:**
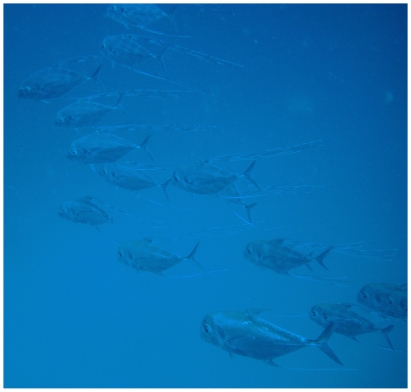
*Alectis ciliaris*, underwater photo by W Toller.

*Alectis ciliaris* (Bloch, 1787)*—*African pompano; **O**; Figure 76

*Caranx bartholomaei* Cuvier, 1833*—*yellow jack; **O, V**

*Caranx crysos* (Mitchill, 1815) *—*blue runner; **O**

*Caranx latus* Agassiz, 1831*—*horse-eye jack; **O, V**

*Caranx lugubris* Poey, 1860*—*black jack; **O, V**

**Figure 77:**
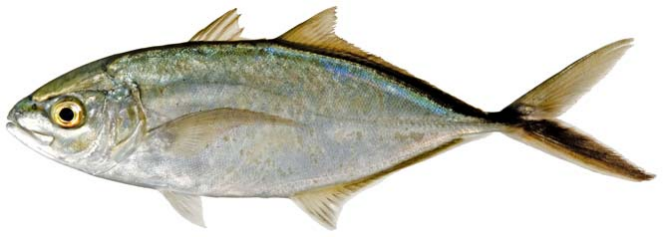
*Caranx ruber*, 260.8 mm SL, photo by JT Williams.

*Caranx ruber* (Bloch, 1793)*—*bar jack; **UF, F, O, V**; Figure 77

**Figure 78:**
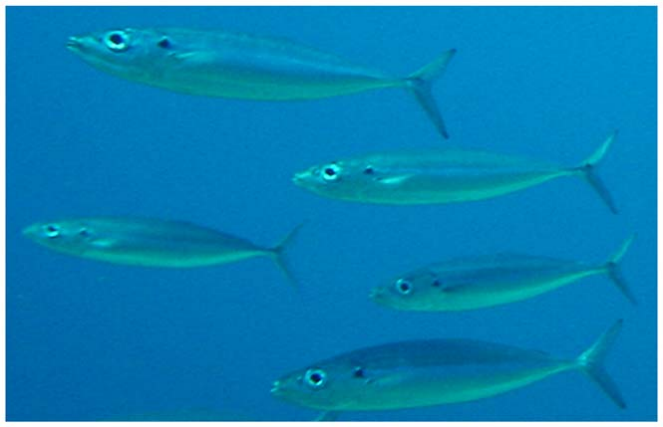
*Decapterus macarellus*, underwater photo by W Toller.

*Decapterus macarellus* (Cuvier, 1833)*—*mackerel scad; **O**; Figure 78

*Elagatis bipinnulata* (Quoy & Gaimard, 1825)*—*rainbow runner; **F, OBS**

**Figure 79:**
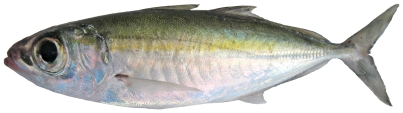
*Selar crumenophthalmus*, 240 mm SL, photo by W Toller.

*Selar crumenophthalmus* (Bloch, 1793)*—*bigeye scad; **F**; Figure 79

**Figure 80:**
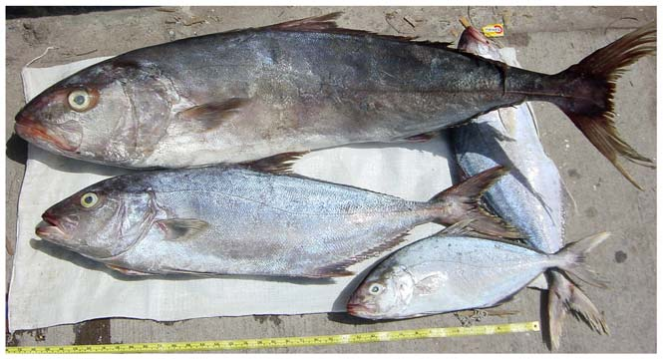
*Seriola dumerili*, large specimen at top; *Seriola rivoliana*, two smaller specimens below, photo by W Toller.

*Seriola dumerili* (Risso, 1810)*—*greater amberjack; **F**; Figure 80

*Seriola rivoliana* Valenciennes, 1833*—*almaco jack; **F**; Figure 80

### Lutjanidae—snappers

**Figure 81:**
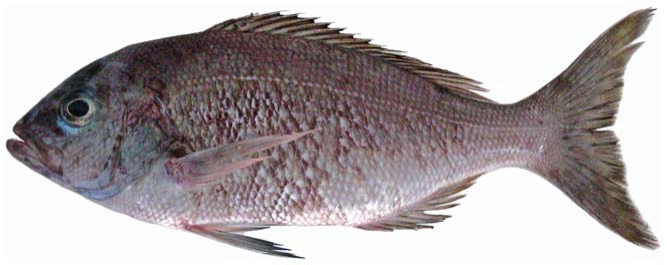
*Apsilus dentatus*, 234 mm SL, photo by W Toller.

*Apsilus dentatus* Guichenot, 1853*—*black snapper; **USNM, F**; Figure 81

*Etelis oculatus* (Valenciennes, 1828) *—*queen snapper; **F**

*Lutjanus apodus* (Walbaum, 1792)*—*schoolmaster; **O, VIS**

**Figure 82:**
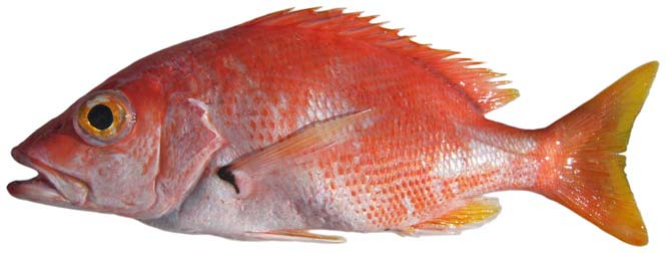
*Lutjanus buccanella*, 249 mm SL, photo by W Toller.

*Lutjanus buccanella* (Cuvier, 1828)*—*blackfin snapper; **USNM, F, O**; Figure 82

*Lutjanus cyanopterus* (Cuvier, 1828)*—*cubera snapper; **USNM, I**

*Lutjanus mahogoni* (Cuvier, 1828) *—*mahogany snapper; **O**

**Figure 83:**
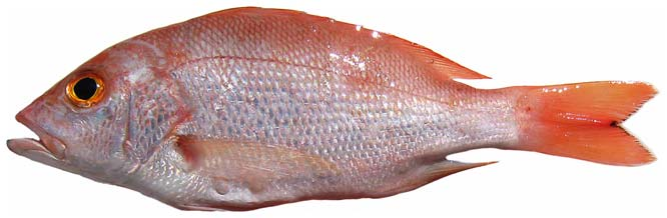
*Lutjanus purpureus*, 207 mm SL, photo by W Toller.

*Lutjanus purpureus* (Poey, 1866)*—*Caribbean red snapper; **USNM, F**; Figure 83

**Figure 84:**
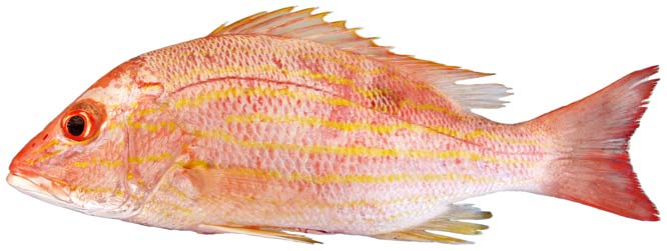
*Lutjanus synagris*, 208 mm SL, photo by W Toller.

*Lutjanus synagris* (Linnaeus, 1758)*—*lane snapper; **USNM, F**; Figure 84

**Figure 85:**
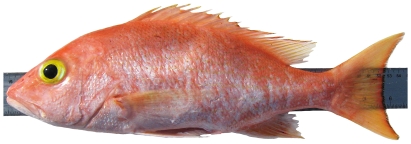
*Lutjanus vivanus*, 311 mm SL, photo by W Toller.

*Lutjanus vivanus* (Cuvier, 1828)*—*silk snapper; **USNM, F**; Figure 85

*Ocyurus chrysurus* (Bloch, 1791)*—*yellowtail snapper; **UF, O, V**

**Figure 86:**
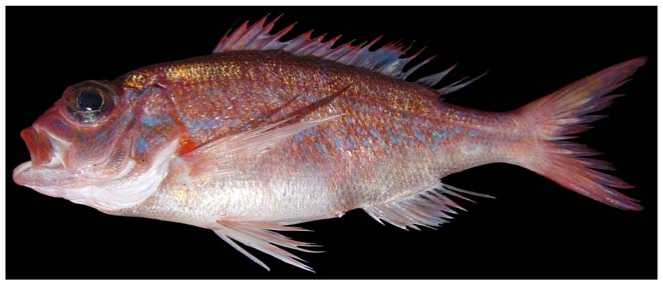
*Pristipomoides aquilonaris*, 263 mm SL, photo by W Toller.

*Pristipomoides aquilonaris* (Goode & Bean, 1896)*—*wenchman; **USNM, F**; Figure 86

**Figure 87:**
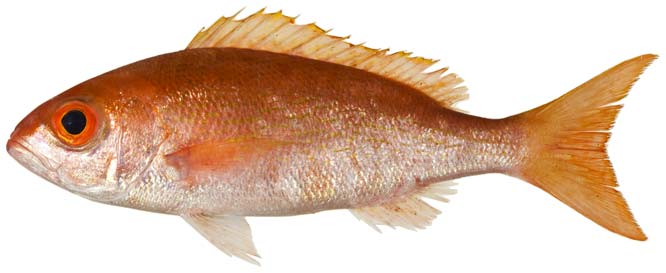
*Rhomboplites aurorubens*, 227.3 mm SL, photo by JT Williams.

*Rhomboplites aurorubens* (Cuvier, 1829) *—*vermilion snapper; **USNM, F**; Figure 87

### Lobotidae—tripletails

**Figure 88:**
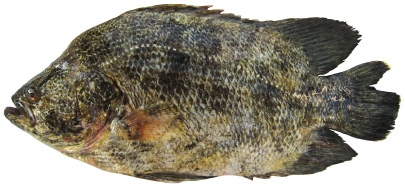
*Lobotes surinamensis*, 300 mm SL, photo by W Toller.

*Lobotes surinamensis* (Bloch, 1790)*—*Atlantic tripletail; **F**; Figure 88

### Haemulidae—grunts

**Figure 89:**
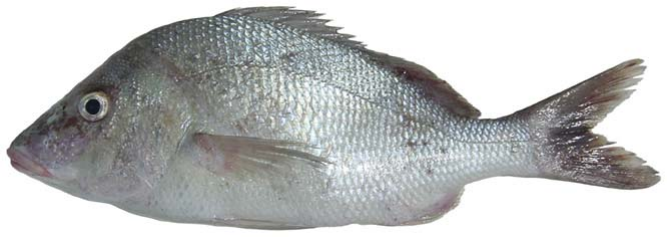
*Haemulon album*, 342 mm SL, photo by W Toller.

*Haemulon album* Cuvier, 1830*—*margate; **F**; Figure 89

**Figure 90:**
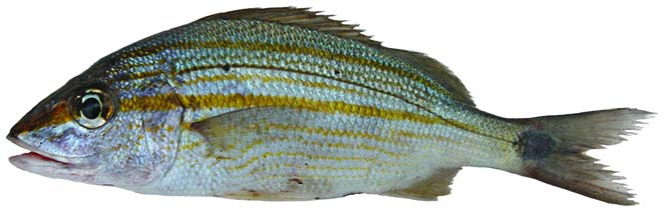
*Haemulon aurolineatum*, 175 mm SL, photo by W Toller.

*Haemulon aurolineatum* Cuvier, 1830*—*tomtate; **F, O**; Figure 90

**Figure 91:**
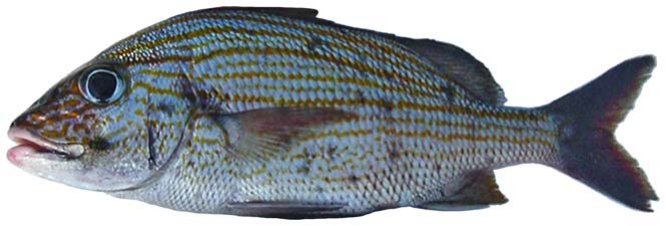
*Haemulon carbonarium*, approximately 200 mm SL, photo by W Toller.

*Haemulon carbonarium* Poey, 1860*—*caesar grunt; **F**; Figure 91

*Haemulon flavolineatum* (Desmarest, 1823)*—*French grunt; **O, V**

**Figure 92:**
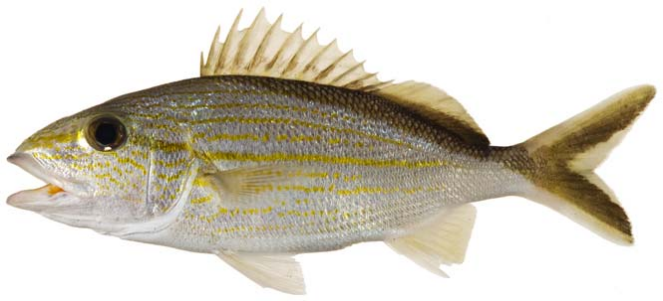
*Haemulon melanurum*, 213.8 mm SL, photo by JT Williams.

*Haemulon melanurum* (Linnaeus, 1758)*—*cottonwick; **UF**, **USNM, F, I, O, V**; Figure 92

**Figure 93:**
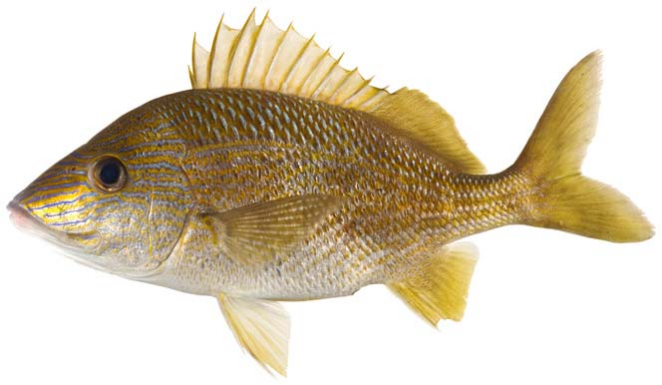
*Haemulon plumierii*, 244.7 mm SL, photo by JT Williams.

*Haemulon plumierii* (Lacepède, 1801)*—*white grunt; **USNM, F, I, O, V**; Figure 93

**Figure 94:**
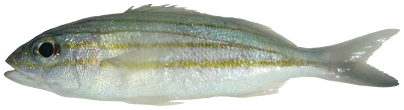
*Haemulon striatum*, 154 mm SL, photo by W Toller.

*Haemulon striatum* (Linnaeus, 1758)*—*striped grunt; **F**; Figure 94

### Inermiidae—bonnetmouths

*Inermia vittata* Poey, 1860*—*boga; **O**

### Sparidae—porgies

*Calamus calamus* (Valenciennes, 1830)*—*saucereye porgy; **V**

### Sciaenidae—drums and croakers

**Figure 95:**
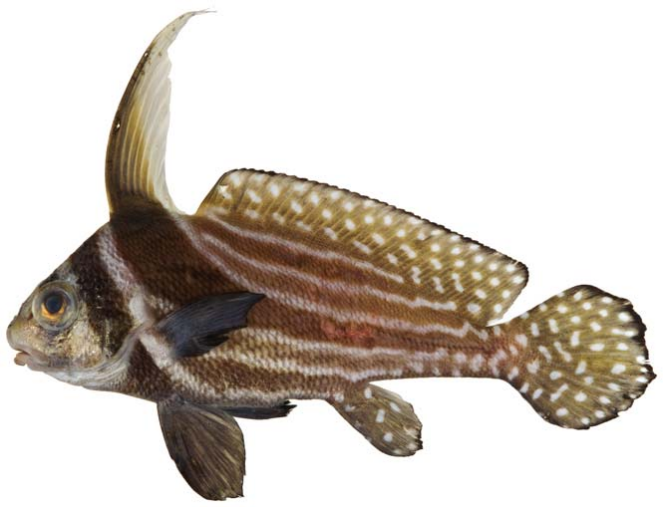
*Equetus punctatus*, 146.8 mm SL, photo by JT Williams.

*Equetus punctatus* (Bloch & Schneider, 1801)*—*spotted drum; **USNM, I**; Figure 95

**Figure 96:**
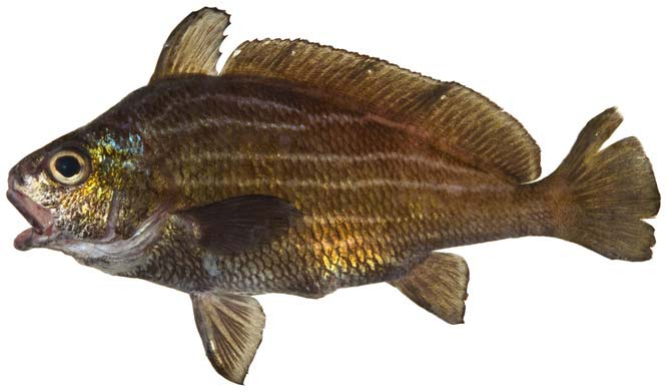
*Pareques acuminatus*, 150.0 mm SL, photo by JT Williams.

*Pareques acuminatus* (Bloch & Schneider, 1801)*—*high-hat; **USNM, I**; Figure 96

### Mullidae—goatfishes

**Figure 97:**
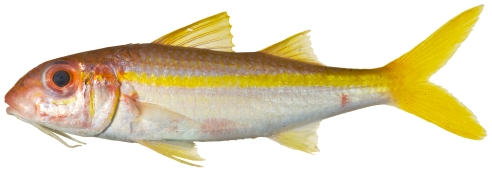
*Mulloidichthys martinicus*, 105 mm SL, photo by JT Williams.

*Mulloidichthys martinicus* (Cuvier, 1829)*—*yellow goatfish; **USNM, F, I, O**; Figure 97

**Figure 98:**
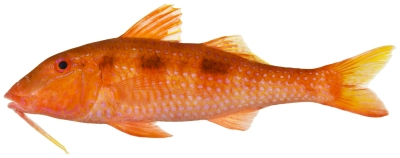
*Pseudupeneus maculatus*, 218.8 mm SL, photo by JT Williams.

*Pseudupeneus maculatus* (Bloch, 1793)*—*spotted goatfish; **USNM, I, O, V**; Figure 98

### Chaetodontidae—butterflyfishes

**Figure 99:**
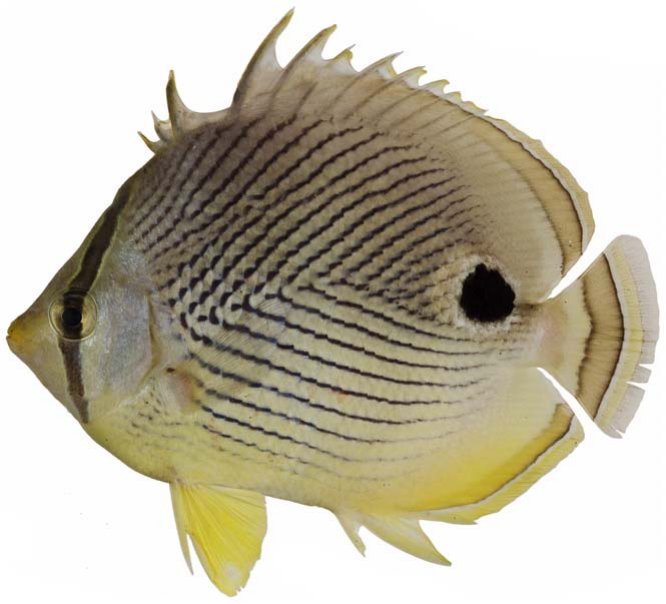
*Chaetodon capistratus*, 99.4 mm SL; when younger, the specimen was apparently injured in the region of the fourth dorsal-fin spine (missing) and the area healed leaving a gap in the fin; photo by JT Williams.

*Chaetodon capistratus* Linnaeus, 1758*—*foureye butterflyfish; **USNM, I, O, V**; Figure 99

*Chaetodon ocellatus* Bloch, 1787*—*spotfin butterflyfish; **USNM, F, O, V**

*Chaetodon sedentarius* Poey, 1860*—*reef butterflyfish; **UF, O, V**

**Figure 100:**
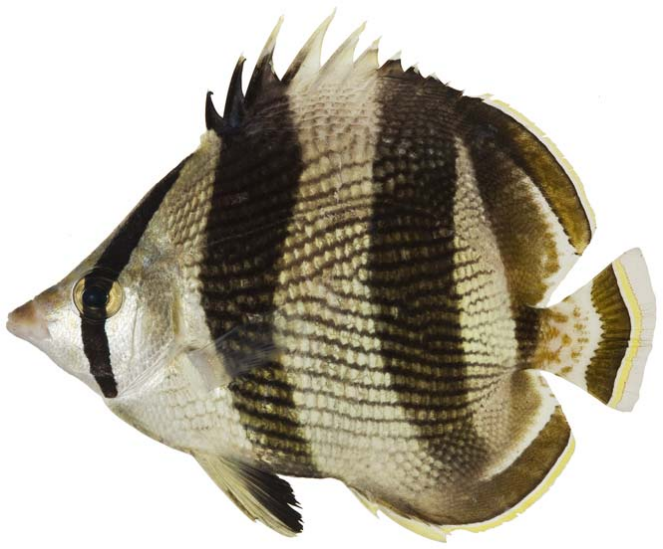
*Chaetodon striatus*, 111.3 mm SL, photo by JT Williams.

*Chaetodon striatus* Linnaeus, 1758*—*banded butterflyfish; **USNM, I, O, V**; Figure 100

**Figure 101:**
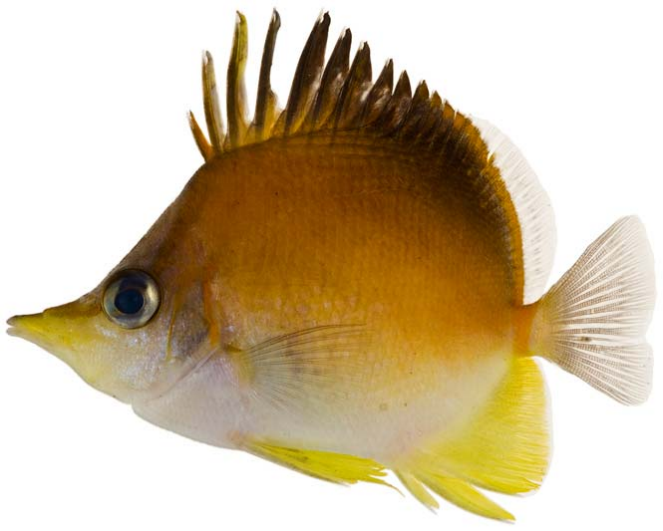
*Prognathodes aculeatus*, 62.9 mm SL, photo by JT Williams.

*Prognathodes aculeatus* (Poey, 1860)*—*longsnout butterflyfish; **USNM, I, O, V**; Figure 101

### Pomacanthidae—angelfishes

**Figure 102:**
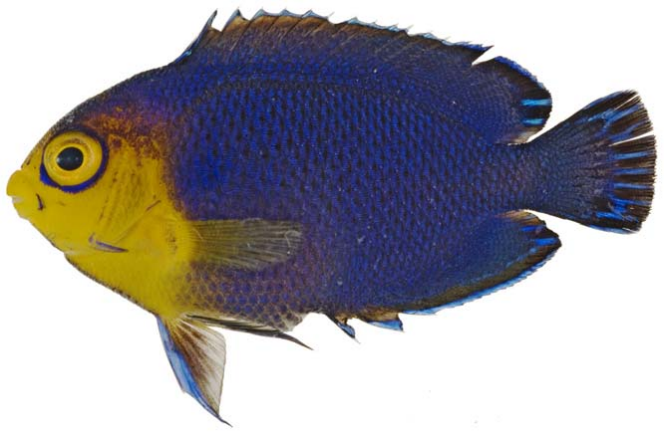
*Centropyge argi*, 33.8 mm SL, photo by JT Williams.

*Centropyge argi* Woods & Kanazawa, 1951*—*cherubfish; **USNM, I, O, V**; Figure 102

*Holacanthus ciliaris* (Linnaeus, 1758)*—*queen angelfish; **USNM, F, I, O, V**

**Figure 103:**
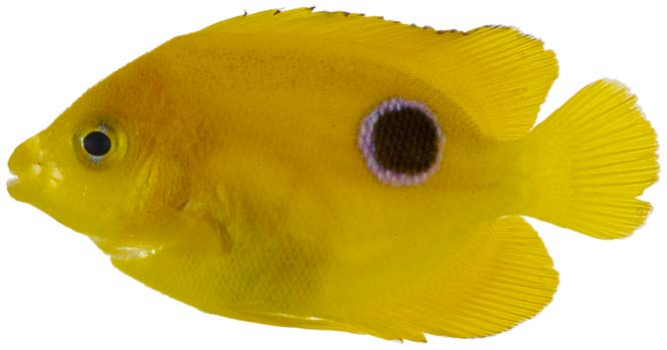
*Holacanthus tricolor*, 19.3 mm SL, juvenile color pattern, photo by JT Williams.

**Figure 104:**
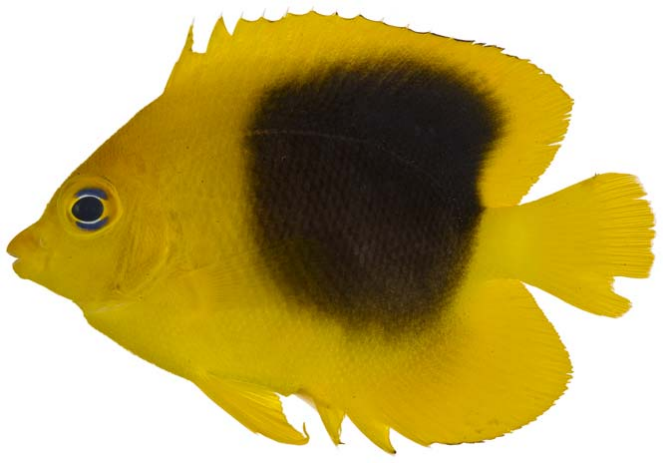
*Holacanthus tricolor*, 40.1 mm SL, intermediate color pattern, photo by JT Williams.

*Holacanthus tricolor* (Bloch, 1795)*—*rock beauty; **USNM, I, O, V**; Figures 103, 104

*Pomacanthus arcuatus* (Linnaeus, 1758)*—*gray angelfish; **V**

*Pomacanthus paru* (Bloch, 1787)*—*French angelfish; **O, V**

### Kyphosidae—sea chub

*Kyphosus incisor* (Cuvier, 1831)*—*yellow chub; **V**

*Kyphosus sectatrix* (Linnaeus, 1766)*—*Bermuda chub; **O, V**

### Cirrhitidae—hawkfishes

**Figure 105:**
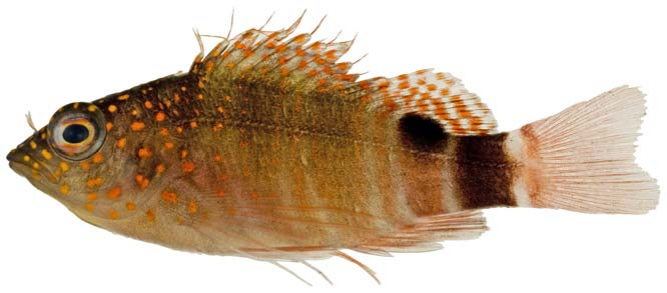
*Amblycirrhitus pinos*, 22.6 mm SL, photo by JT Williams.

*Amblycirrhitus pinos* (Mowbray, 1927)*—*redspotted hawkfish; **USNM, I, O**; Figure 105

### Pomacentridae-damselfishes

**Figure 106:**
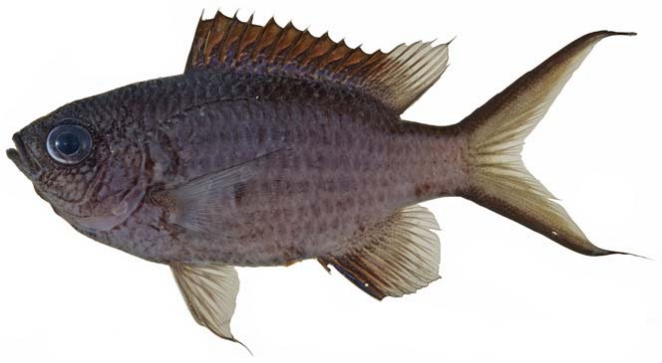
*Chromis cyanea*, 67.5 mm SL, iridescent blue colors on body faded immediately after death, photo by JT Williams.

*Chromis cyanea* (Poey, 1860)*—*blue chromis; **USNM, I, O, V**; Figure 106

**Figure 107:**
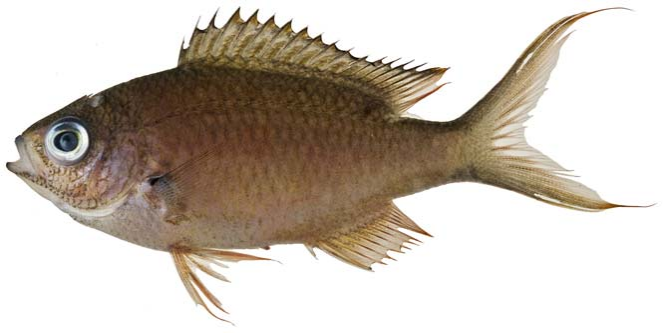
*Chromis multilineata*, 56.3 mm SL, photo by JT Williams.

*Chromis multilineata* (Guichenot, 1853)*—*brown chromis; **USNM, I, O, V**; Figure 107

*Microspathodon chrysurus* (Cuvier, 1830)*—*yellowtail damselfish; **O, V**

*Stegastes adustus* (Troschel, 1865) *—*dusky damselfish; **O**

*Stegastes leucostictus* (Müller & Troschel, 1848) *—*beaugregory; **O**

**Figure 108:**
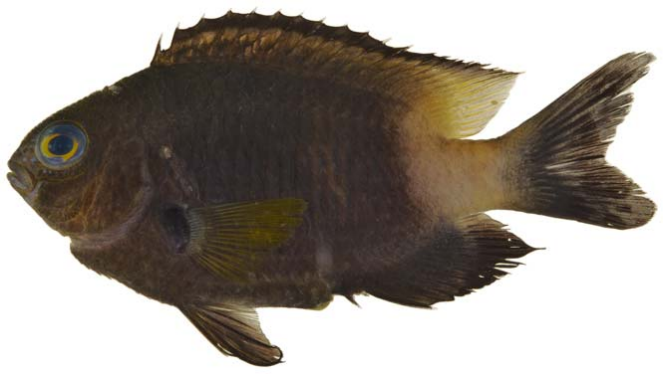
*Stegastes partitus*, 58.6 mm SL, black-tailed color morph, photo by JT Williams.

**Figure 109:**
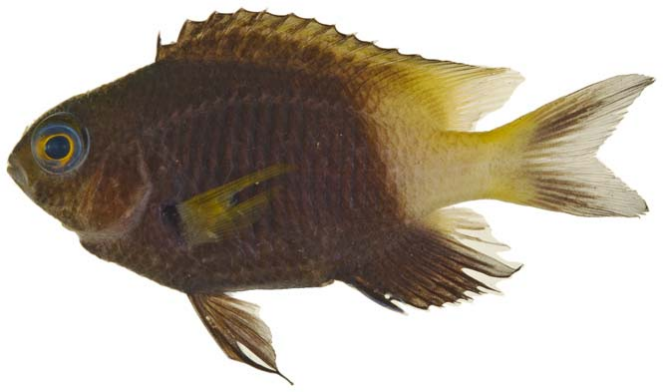
*Stegastes partitus*, 58.6 mm SL, yellow-tailed color morph, photo by JT Williams.

*Stegastes partitus* (Poey, 1868)*—*bicolor damselfish; **USNM, I, O, V**; Figures 108, 109

This species has a variable color pattern throughout its range. Two color morphs were found at Saba Bank. Both morphs have a yellow pectoral fin and a reduced yellowish white area covering the caudal peduncle. One morph has a black caudal fin (Figure 108) and the other morph has a pale yellowish caudal fin with a dusky brown area in the middle of the upper and lower lobes (Figure 109).

**Figure 110:**
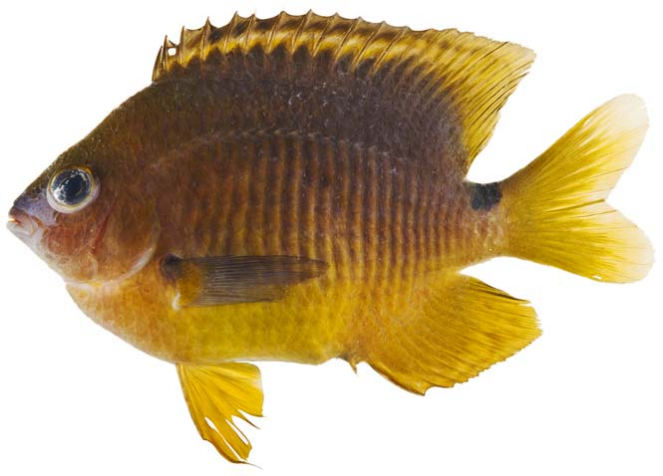
*Stegastes planifrons*, 82.7 mm SL, photo by JT Williams.

*Stegastes planifrons* (Cuvier, 1830)*—*threespot damselfish; **USNM, I, O**; Figure 110

### Labridae—wrasses

*Bodianus rufus* (Linnaeus, 1758)*—*Spanish hogfish; **O, V**

**Figure 111:**
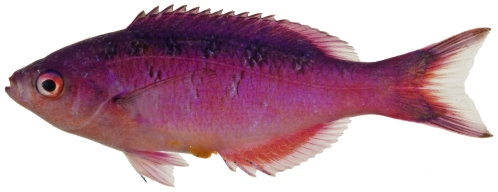
*Clepticus parrae*, 59.8 mm SL, photo by JT Williams.

*Clepticus parrae* (Bloch & Schneider, 1801)*—*creole wrasse; **USNM, I, O, V**; Figure 111

**Figure 112:**
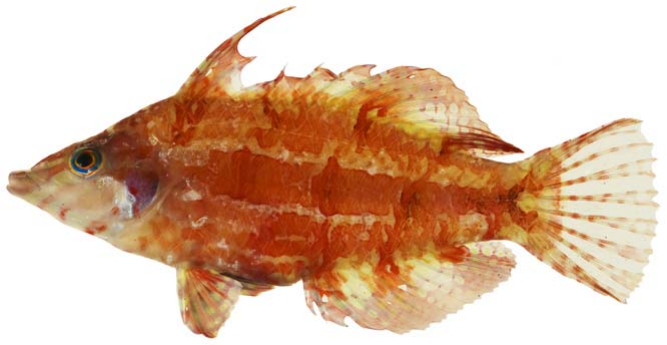
*Doratonotus megalepis*, 44.1 mm SL, photo by JT Williams.

*Doratonotus megalepis* Günther, 1862*—*dwarf wrasse; **USNM, I, V**; Figure 112

**Figure 113:**
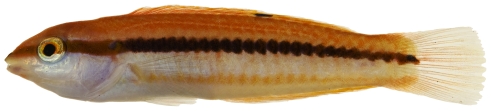
*Halichoeres bivittatus*, 37.9 mm SL, juvenile/initial color phase, showing the black spot in the dorsal fin and a somewhat unusual orangish anal fin, photo by JT Williams.

**Figure 114:**
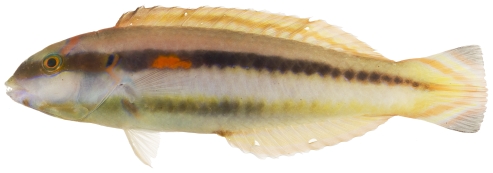
*Halichoeres bivittatus*, 103.0 mm SL, terminal male color phase, with a distinctive red blotch on the side of the body above the pectoral fin, photo by JT Williams.

*Halichoeres bivittatus* (Bloch, 1791)*—*slippery dick; **USNM, I, O, V**; Figures 113, 114

*Halichoeres cyanocephalus* (Bloch, 1791)*—*yellowcheek wrasse; **O, V**

**Figure 115:**
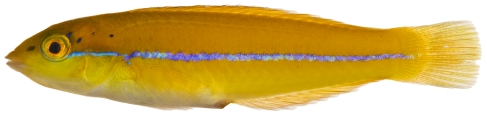
*Halichoeres garnoti*, 46.4 mm SL, juvenile color phase, showing the characteristic blue stripe on a yellow body, photo by JT Williams.

**Figure 116:**
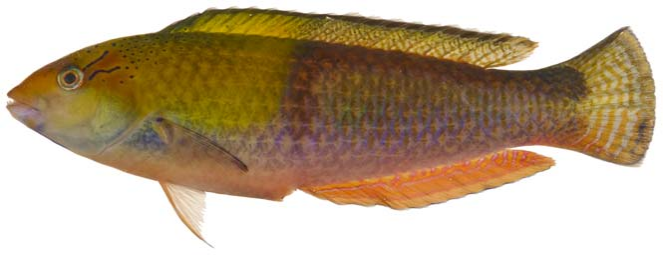
*Halichoeres garnoti*, 118.1 mm SL, initial/terminal color phase, this specimen has almost completed the transition from the female initial phase into a terminal male, photo by JT Williams.

**Figure 117:**
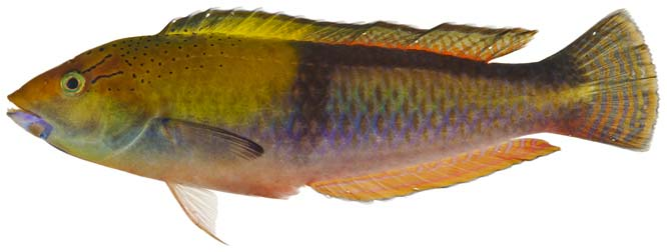
*Halichoeres garnoti*, 125.3 mm SL, terminal male color phase, this specimen has completed the transition from the female initial phase into a terminal male and shows the characteristic black bar and dark area over the caudal peduncle, photo by JT Williams.

*Halichoeres garnoti* (Valenciennes, 1839)*—*yellowhead wrasse; **UF**, **USNM, I, O, V**; Figures 115, 116, 117

These figures show portions of the transitional color phases as individuals transform from juveniles (Figure 115) into initial phase females (Figure 116) and finally into terminal phase males (Figure 117).

*Halichoeres maculipinna* (Müller & Troschel, 1848)*—*clown wrasse; **USNM, I, O**

**Figure 118:**
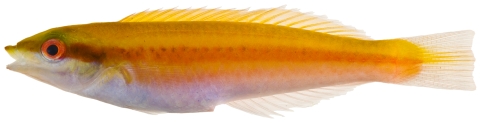
*Halichoeres pictus*, 70.7 mm SL, juvenile color phase, photo by JT Williams.

**Figure 119:**
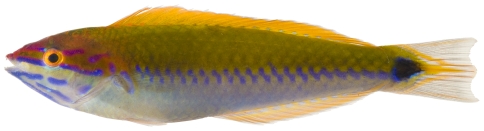
*Halichoeres pictus*, 85.0 mm SL, terminal male color phase, photo by JT Williams.

*Halichoeres pictus* (Poey, 1860)*—*rainbow wrasse; **USNM, I, O**; Figures 118, 119

**Figure 120:**
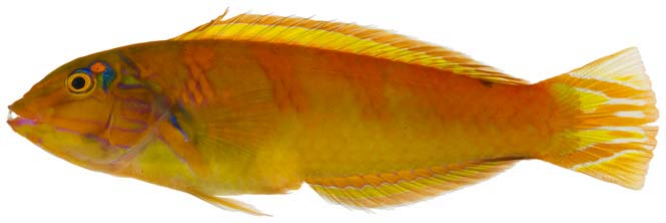
*Halichoeres poeyi*, 87.9 mm SL, terminal male color phase, photo by JT Williams.

*Halichoeres poeyi* (Steindachner, 1867)*—*blackear wrasse; **UF**, **USNM, I, O, V**; Figure 120

*Halichoeres radiatus* (Linnaeus, 1758)*—*puddingwife; **O, V**

*Lachnolaimus maximus* (Walbaum, 1792)*—*hogfish; **USNM**

**Figure 121:**
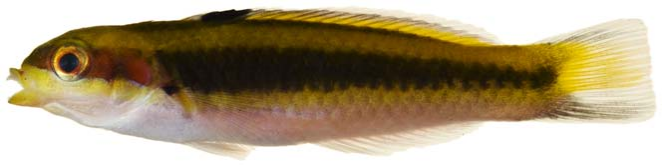
*Thalassoma bifasciatum*, 39.3 mm SL, juvenile/initial color phase, photo by JT Williams.

**Figure 122:**
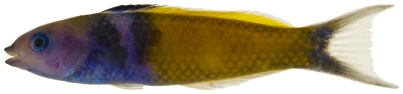
*Thalassoma bifasciatum*, 63.5 mm SL, terminal male color phase, photo by JT Williams.

*Thalassoma bifasciatum* (Bloch, 1791)*—*bluehead; **USNM, I, O, V**; Figures 121, 122

**Figure 123:**
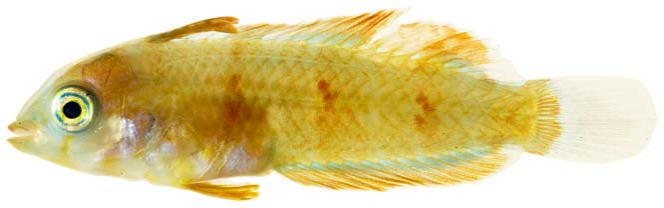
*Xyrichtys splendens*, 15.1 mm SL, young juvenile color phase, photo by JT Williams.

**Figure 124:**
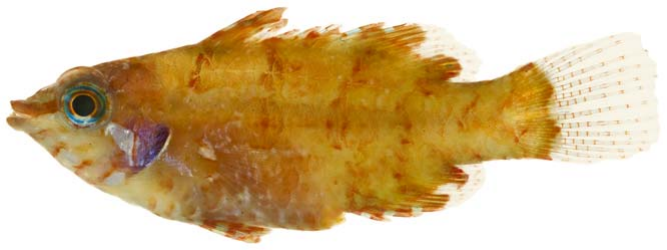
*Xyrichtys splendens*, 22.1 mm SL, juvenile color phase, photo by JT Williams.

**Figure 125:**
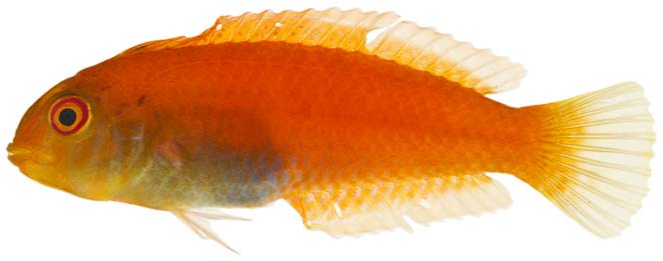
*Xyrichtys splendens*, 52.4 mm SL, initial color phase of female, photo by JT Williams.

**Figure 126:**
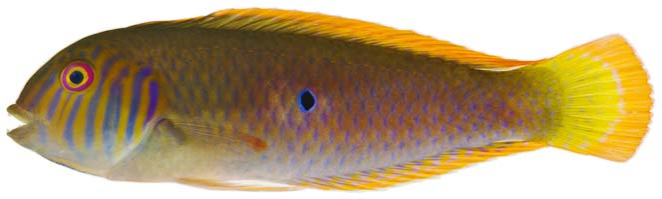
*Xyrichtys splendens*, 72.5 mm SL, terminal male color phase, photo by JT Williams.

*Xyrichtys splendens* Castelnau, 1855*—*green razorfish; **USNM, I, O**; Figures 123, 124, 125, 126

### Scaridae—parrotfishes

**Figure 127:**
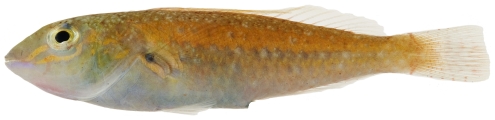
*Cryptotomus roseus*, 61.5 mm SL, terminal male color phase, photo by JT Williams.

*Cryptotomus roseus* Cope, 1871*—*bluelip parrotfish; **UF**, **USNM, I, O**; Figure 127

*Scarus coelestinus* Valenciennes, 1840*—*midnight parrotfish; **V**

**Figure 128:**
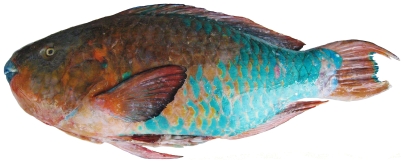
*Scarus guacamaia*, 647 mm SL, terminal male color phase, photo by W Toller.

*Scarus guacamaia* Cuvier, 1829*—*rainbow parrotfish; **F**; Figure 128

*Scarus iseri* (Bloch, 1789)*—*striped parrotfish; **USNM, O, V**

**Figure 129:**
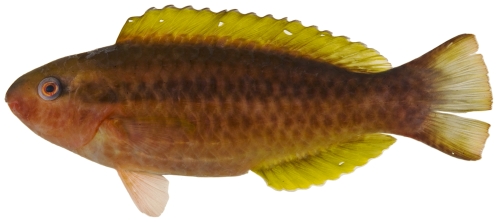
*Scarus taeniopterus*, 174.9 mm SL, initial female color phase, photo by JT Williams.

**Figure 130:**
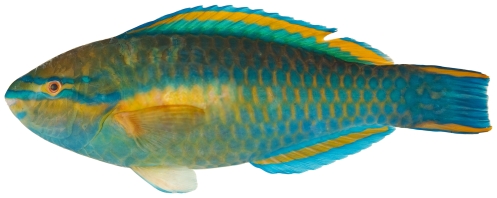
*Scarus taeniopterus*, 231.7 mm SL, terminal male color phase, photo by JT Williams.

*Scarus taeniopterus* Desmarest, 1831*—*princess parrotfish; **USNM, I, O, V**; Figure 129, 130

*Scarus vetula* Bloch & Schneider, 1801*—*queen parrotfish; **O**

**Figure 131:**
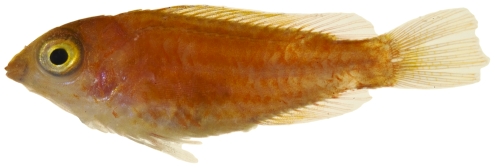
*Sparisoma atomarium*, 13.7 mm SL, juvenile color phase, photo by JT Williams.

*Sparisoma atomarium* (Poey, 1861)*—*greenblotch parrotfish; **USNM, I, O**; Figure 131

**Figure 132:**
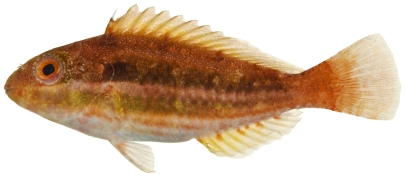
*Sparisoma aurofrenatum*, 53.8 mm SL, juvenile color phase, photo by JT Williams.

**Figure 133:**
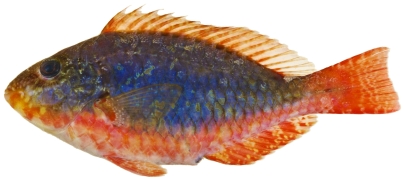
*Sparisoma aurofrenatum*, 164.0 mm SL, initial female color phase, photo by JT Williams.

**Figure 134:**
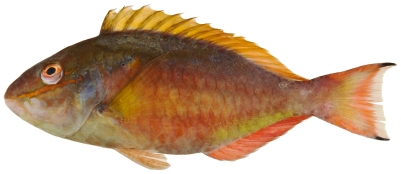
*Sparisoma aurofrenatum*, 164 mm SL, terminal male color phase, photo by JT Williams.

*Sparisoma aurofrenatum* (Valenciennes, 1840)*—*redband parrotfish; **USNM, I, O, V**; Figures 132, 133, 134

**Figure 135:**
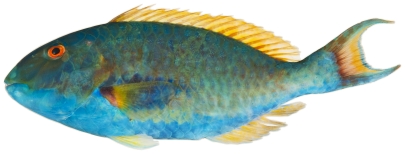
*Sparisoma chrysopterum*, 274 mm SL, terminal male color phase, photo by JT Williams.

*Sparisoma chrysopterum* (Bloch & Schneider, 1801)*—*redtail parrotfish; **USNM, I, O**; Figure 135

**Figure 136:**
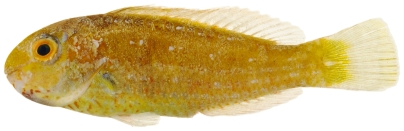
*Sparisoma radians*, 51.6 mm SL, juvenile color phase, photo by JT Williams.

*Sparisoma radians* (Valenciennes, 1840)*—*bucktooth parrotfish; **USNM, I, O, V**; Figure 136

**Figure 137:**
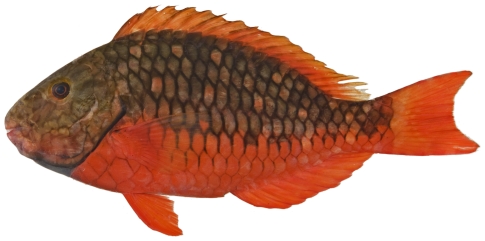
*Sparisoma viride*, 222.9 mm SL, initial female color phase, photo by JT Williams.

*Sparisoma viride* (Bonnaterre, 1788)*—*stoplight parrotfish; **USNM, I, O, V**; Figure 137

### Tripterygiidae—triplefins

**Figure 138:**
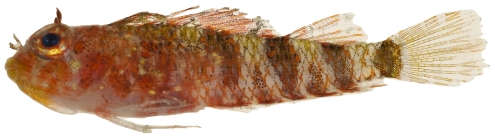
*Enneanectes altivelis*, 18.3 mm SL, photo by JT Williams.

*Enneanectes altivelis* Rosenblatt, 1960 *—*lofty triplefin; **USNM, I**; Figure 138

*Enneanectes atrorus* Rosenblatt, 1960*—*redeye triplefin; **USNM, I**

*Enneanectes jordani* (Evermann and Marsh, 1899) *—*mimic triplefin; **USNM, I**

### Dactyloscopidae-sand stargazers

**Figure 139:**
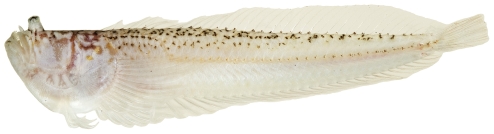
*Dactyloscopus tridigitatus*, 50.8 mm SL, photo by JT Williams.

**Figure 140:**
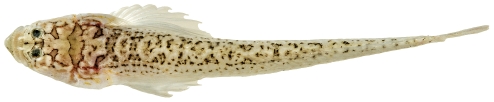
*Dactyloscopus tridigitatus*, 50.8 mm SL, dorsal view, photo by JT Williams.

*Dactyloscopus tridigitatus* Gill, 1859*—*sand stargazer; **USNM, I**; Figures 139, 140

**Figure 141:**
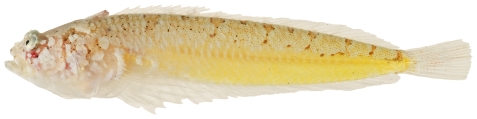
*Gillellus uranidea*, 27.7 mm SL, photo by JT Williams.

*Gillellus uranidea* Böhlke, 1968*—*warteye stargazer; **USNM, I**; Figure 141

**Figure 142:**
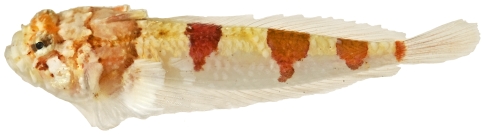
*Platygillellus rubrocinctus*, 24.1 mm SL, adult, photo by JT Williams.

**Figure 143:**
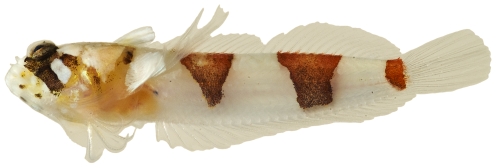
*Platygillellus rubrocinctus*, 12.1 mm SL, lateral view of juvenile, photo by JT Williams.

**Figure 144:**
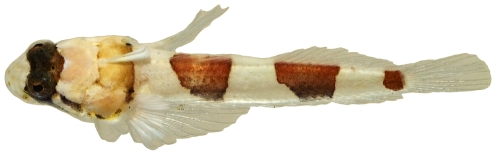
*Platygillellus rubrocinctus*, 12.1 mm SL, dorsal view of juvenile, photo by JT Williams.

*Platygillellus rubrocinctus* (Longley, 1934)*—*saddle stargazer; **USNM, I**; Figures 142, 143, 144

### Blenniidae—combtooth blennies

*Parablennius marmoreus* (Poey, 1876)*—*seaweed blenny; **USNM, I**

### Labrisomidae—scaly blennies

**Figure 145:**
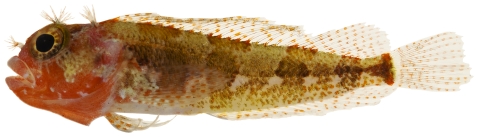
*Labrisomus gobio*, 36.2 mm SL, photo by JT Williams.

*Labrisomus gobio* (Valenciennes, 1836)*—*palehead blenny; **USNM, I**; Figure 145

**Figure 146:**
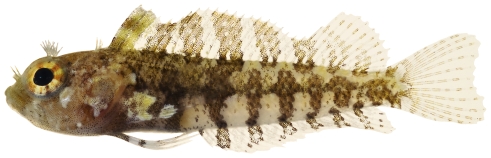
*Labrisomus haitiensis*, 18.8 mm SL, photo by JT Williams.

*Labrisomus haitiensis* Beebe &Tee-Van, 1918*—*longfin blenny; **USNM, I**; Figure 146

**Figure 147:**
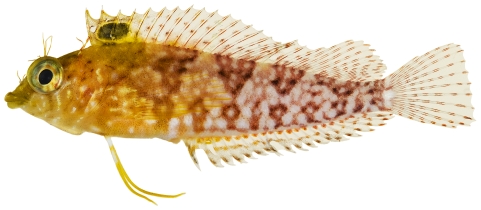
*Malacoctenus boehlkei*, 36.3 mm SL, photo by JT Williams.

*Malacoctenus boehlkei* Springer, 1959*—*diamond blenny; **USNM, I**; Figure 147

**Figure 148:**
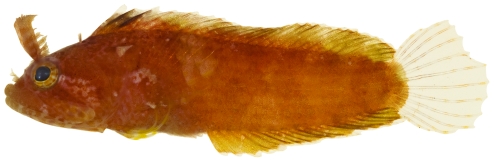
*Paraclinus grandicomis*, 27.1 mm SL, photo by JT Williams.

*Paraclinus grandicomis* (Rosen, 1911)*—*horned blenny; **UF**, **USNM, I**; Figure 148

**Figure 149:**
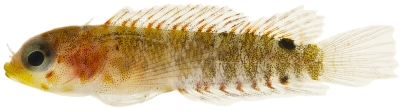
*Starksia atlantica*, 9.5 mm SL, juvenile, photo by JT Williams.

**Figure 150:**
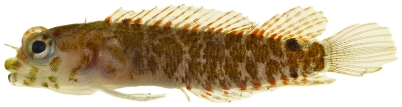
*Starksia atlantica*, 15.1 mm SL, adult male, photo by JT Williams.

*Starksia atlantica* Longley, 1934*—*smootheye blenny; **USNM, I**; Figures 149, 150

The Saba Bank population may be a distinct species in the species complex currently referred to as *Starksia atlantica*. This complex requires additional taxonomic study.

**Figure 151:**
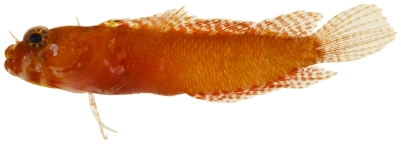
*Starksia lepicoelia*, 20.2 mm SL, adult female, photo by JT Williams.

**Figure 152:**
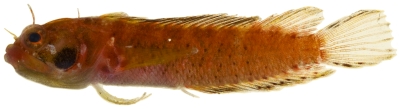
*Starksia lepicoelia*, 19.8 mm SL, adult male, photo by JT Williams.

*Starksia* cf *lepicoelia* Böhlke & Springer, 1961*—*blackcheek blenny; **USNM, I**; Figures 151, 152

Females (Figure 151) lack the black spot on the cheek that is characteristic of mature males (Figure 152). The Saba Bank population may be a distinct species in the species complex currently referred to as *Starksia lepicoelia*. This complex requires additional taxonomic study.

*Starksia melasma* Williams & Mounts, 2003*—*black spot blenny; **USNM, I**

**Figure 153:**
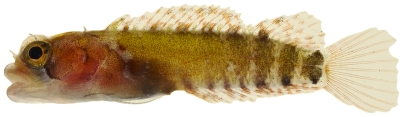
*Starksia nanodes*, 10.6 mm SL, adult male, photo by JT Williams.

*Starksia nanodes* Böhlke & Springer, 1961*—*dwarf blenny; **USNM, I**; Figure 153

The Saba Bank population may be a distinct species in the species complex currently referred to as *Starksia nanodes*. This complex requires additional taxonomic study.

### Chaenopsidae—tube blennies

**Figure 154:**
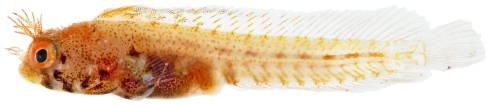
*Acanthemblemaria aspera*, 14.0 mm SL, juvenile/female, photo by JT Williams.

**Figure 155:**
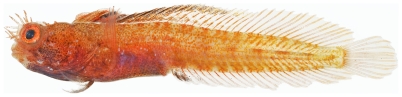
*Acanthemblemaria aspera*, 19.7 mm SL, adult male, photo by JT Williams.

**Figure 156:**
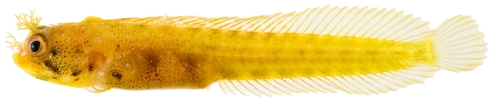
*Acanthemblemaria aspera*, 20.4 mm SL, yellow color morph (female?), photo by JT Williams.

*Acanthemblemaria aspera* (Longley, 1927)*—*roughhead blenny; **USNM, I**; Figures 154, 155, 156

Three different color patterns were observed at Saba Bank: juvenile/female (Figure 154), adult male (Figure 155), and a distinctive yellow, probably female, color morph (Figure 156). The adult male and the yellow morph were taken together at the same collecting station.

**Figure 157:**
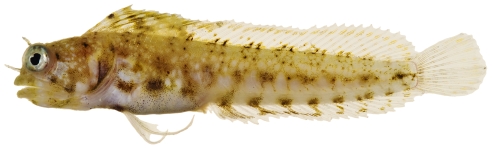
*Emblemaria pandionis*, 37.0 mm SL, adult female, photo by JT Williams.

*Emblemaria pandionis* Evermann & Marsh, 1900*—*sailfin blenny; **USNM, I**; Figure 157

**Figure 158:**
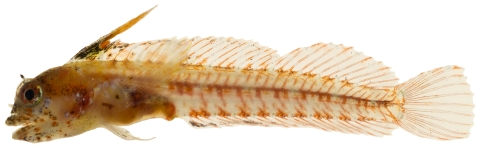
*Emblemariopsis* cf *signifer*, 16.2 mm SL, adult male, this specimen represents an undescribed species in the *signifer* species complex, photo by JT Williams.

*Emblemariopsis* cf *signifer* (Ginsburg, 1942)*—*flagfin blenny; **USNM, I**; Figure 158

The *signifer* species complex ranges from Brazil throughout the Caribbean and includes a number of undescribed species in the Caribbean region. Additional taxonomic study is required to resolve the taxa.

### Gobiesocidae—clingfishes

**Figure 159:**
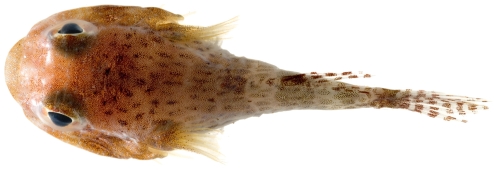
*Acyrtus artius*, 20.4 mm SL, dorsal view, photo by JT Williams.

**Figure 160:**
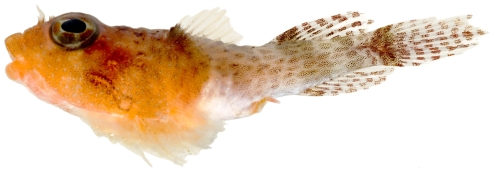
*Acyrtus artius*, 20.4 mm SL, lateral view, photo by JT Williams.

**Figure 161:**
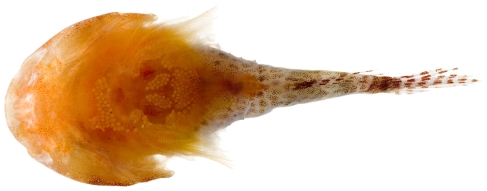
*Acyrtus artius*, 20.4 mm SL, ventral view showing pelvic disk, photo by JT Williams.

*Acyrtus artius* Briggs, 1955*—*papillate clingfish; **USNM, I**; Figures 159, 160, 161

### Callionymidae—dragonets

**Figure 162:**
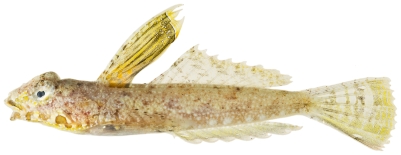
*Paradiplogrammus bairdi*, 39.8 mm SL, lateral view of an adult male, photo by JT Williams.

**Figure 163:**
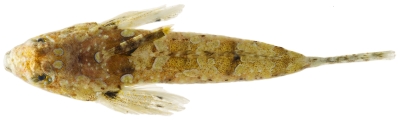
*Paradiplogrammus bairdi*, 29.8 mm SL, dorsal view of a smaller adult male, photo by JT Williams.

*Paradiplogrammus bairdi* Jordan, 1888*—*lancer dragonet; **USNM, I, O**; Figures 162, 163

### Gobiidae-gobies

**Figure 164:**
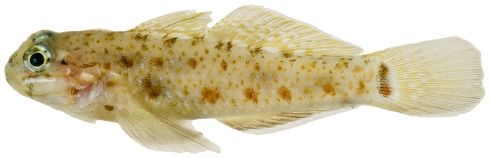
*Coryphopterus dicrus*, 31.8 mm SL, photo by JT Williams.

*Coryphopterus dicrus* Böhlke & Robins, 1960*—*colon goby; **USNM, I**; Figure 164

**Figure 165:**
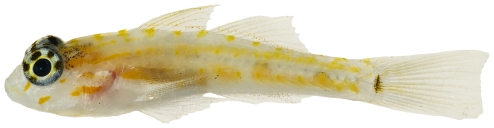
*Coryphopterus eidolon*, 18.3 mm SL, photo by JT Williams.

*Coryphopterus eidolon* Böhlke & Robins, 1960*—*pallid goby; **USNM, I**; Figure 165

**Figure 166:**
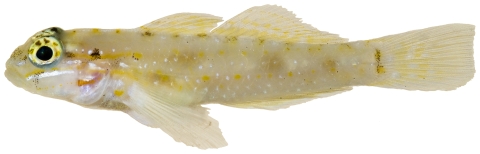
*Coryphopterus glaucofraenum*, 33.7 mm SL, photo by JT Williams.

*Coryphopterus glaucofraenum* Gill, 1863*—*bridled goby; **USNM, I, V**; Figure 166

**Figure 167:**
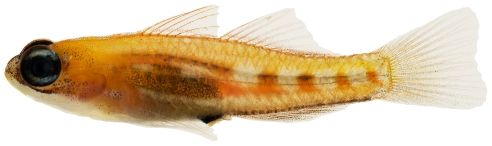
*Coryphopterus personatus*, 19.0 mm SL, photo by JT Williams.

*Coryphopterus personatus* (Jordan & Thompson, 1905)*—*masked goby; **USNM, I**; Figure 167

**Figure 168:**
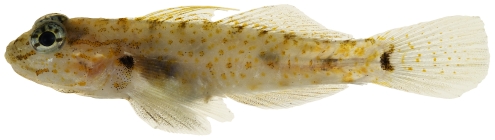
*Coryphopterus thrix*, 26.8 mm SL, photo by JT Williams.

*Coryphopterus thrix* Böhlke & Robins, 1960*—*bartail goby; **USNM, I**; Figure 168

**Figure 169:**
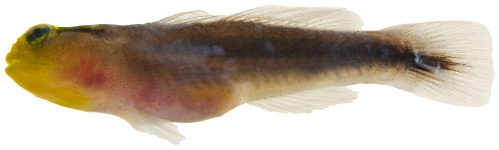
*Elacatinus chancei*, 28.1 mm SL, photo by JT Williams.

*Elacatinus chancei* (Beebe & Hollister 1933)*—*shortstripe goby; **USNM, I**; Figure 169

**Figure 170:**
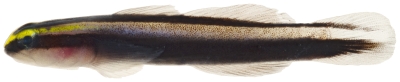
*Elacatinus evelynae*, 27.7 mm SL, photo by JT Williams.

*Elacatinus evelynae* (Böhlke & Robins, 1968)*—*sharknose goby; **USNM, I**; Figure 170

*Elacatinus genie* (Böhlke & Robins, 1968)*—*cleaner goby; **USNM, I**

**Figure 171:**
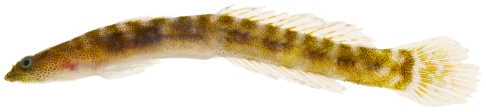
*Evermannichthys metzelaari*, 21.0 mm SL, photo by JT Williams.

*Evermannichthys metzelaari* Hubbs, 1923 *—*roughtail goby; **USNM, I**; Figure 171

**Figure 172:**
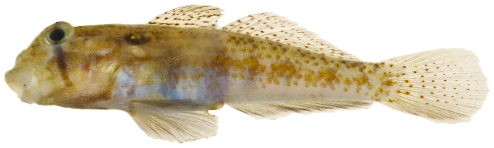
*Gnatholepis thompsoni*, 31.5 mm SL, photo by JT Williams.

*Gnatholepis thompsoni* Jordan, 1904*—*goldspot goby; **USNM, I, V**; Figure 172

**Figure 173:**
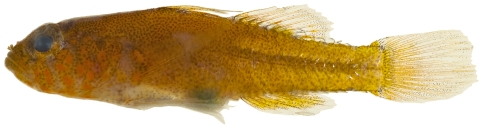
*Lythrypnus elasson*, 9.2 mm SL, adult male, photo by JT Williams.

*Lythrypnus elasson* Böhlke & Robins, 1960*—*dwarf goby; **USNM, I**; Figure 173

**Figure 174:**
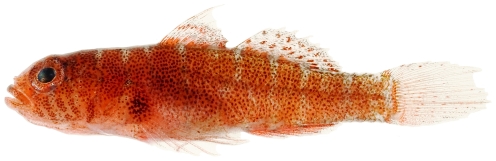
*Lythrypnus minimus*, 10.6 mm SL, adult male, photo by JT Williams.

*Lythrypnus minimus* Garzón & Acero P., 1988 *—*pygmy goby; **USNM, I**; Figure 174

**Figure 175:**
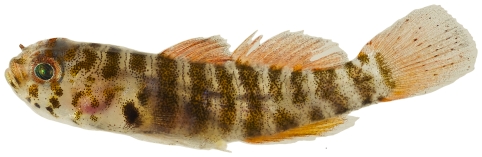
*Lythrypnus nesiotes*, 11.9 mm SL, adult male, photo by JT Williams.

*Lythrypnus nesiotes* Böhlke & Robins, 1960*—*island goby; **USNM, I**; Figure 175

**Figure 176:**
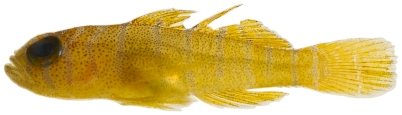
*Priolepis hipoliti*, 12.2 mm SL, female, photo by JT Williams.

**Figure 177:**
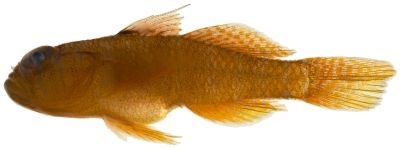
*Priolepis hipoliti*, 18.7 mm SL, adult male, photo by JT Williams.

*Priolepis hipoliti* (Metzelaar, 1922)*—*rusty goby; **USNM, I**; Figures 176, 177

**Figure 178:**
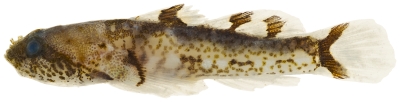
*Psilotris batrachodes*, 13.3 mm SL, adult, photo by JT Williams.

*Psilotris batrachodes* Böhlke, 1963*—*toadfish goby; **USNM, I**; Figure 178

**Figure 179:**
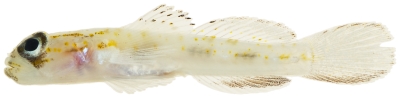
*Psilotris boehlkei*, 26.5 mm SL, adult, photo by JT Williams.

*Psilotris boehlkei* Greenfield, 1993*—*yellowspot goby; **I**; Figure 179

The fresh colors of *P. boehlkei* are presented (Figure 179) for the first time. This very rare species was previously known from only five specimens taken at St. Barthelemey in 1965. The single specimen we collected extends the known distribution of *P. boehlkei* to Saba Bank. We have named this species the yellowspot goby in reference to the yellow spots on the head and body.

**Figure 180:**
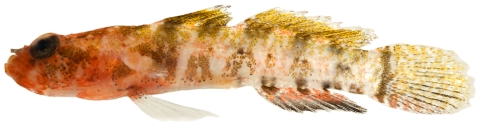
*Pycnomma roosevelti*, 14.6 mm SL, adult, photo by JT Williams.

*Pycnomma roosevelti* Ginsburg, 1939 *—*Roosevelt's goby; **USNM, I**; Figure 180

Although *P. roosevelti* has previously been taken from a several scattered localities around the Caribbean (Isla Providencia, Guadeloupe, Belize and Puerto Rico), there are fewer than 10 specimens known and its fresh colors (Figure 180) have not been published previously.

**Figure 181:**
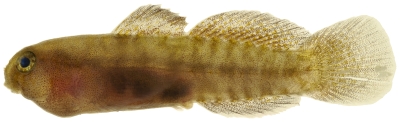
*Risor ruber*, 18.7 mm SL, adult, photo by JT Williams.

*Risor ruber* (Rosén, 1911) *—*tusked goby; **USNM, I**; Figure 181

### Acanthuridae—surgeonfishes

**Figure 182:**
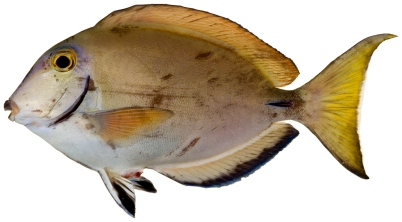
*Acanthurus bahianus*, 128.2 mm SL, photo by JT Williams.

*Acanthurus bahianus* Castelnau, 1855*—*ocean surgeon; **USNM, I, O, V**; Figure 182

**Figure 183:**
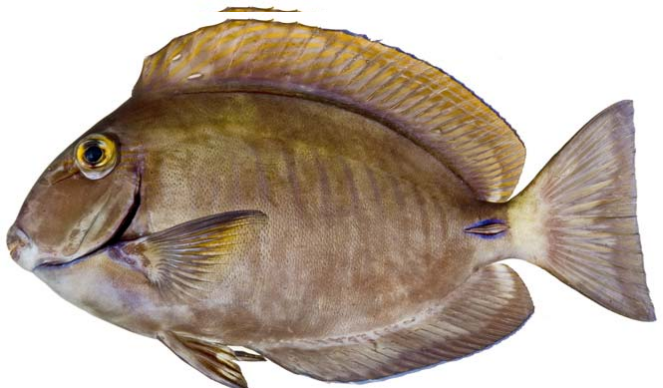
*Acanthurus chirurgus*, 168.0 mm SL, photo by JT Williams.

*Acanthurus chirurgus* (Bloch, 1787)*—*doctorfish; **USNM, I, O, V**; Figure 183

**Figure 184:**
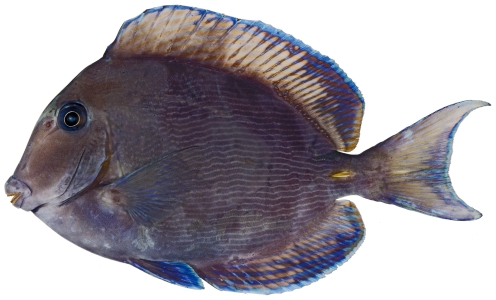
*Acanthurus coeruleus*, 146.2 mm SL, photo by JT Williams.

*Acanthurus coeruleus* Bloch & Schneider, 1801*—*blue tang; **USNM, I, O, V**; Figure 184

### Sphyraenidae—barracudas

*Sphyraena barracuda* (Walbaum, 1792)*—*great barracuda; **O, V**

### Scombridae—mackerels

*Acanthocybium solandri* (Cuvier, 1832)—wahoo; **F**

**Figure 185:**
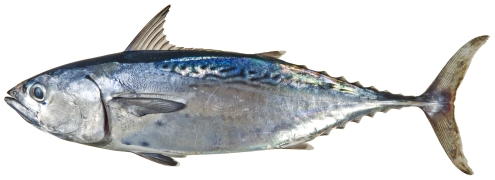
*Euthynnus alletteratus*, 355.1 mm FL, photo by JT Williams.

*Euthynnus alletteratus* (Rafinesque, 1810)*—*little tunny; **USNM**; Figure 185

*Scomberomorus regalis* (Bloch, 1793)*—*cero; **USNM**

**Figure 186:**
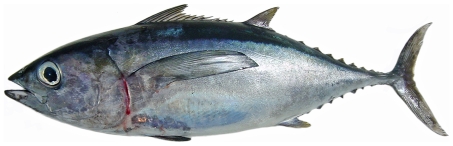
*Thunnus atlanticus*, 516 mm FL, photo by W Toller.

*Thunnus atlanticus* (Lesson 1831)*—*blackfin tuna; **F**; Figure 186

### Caproidae—boarfishes

**Figure 187:**
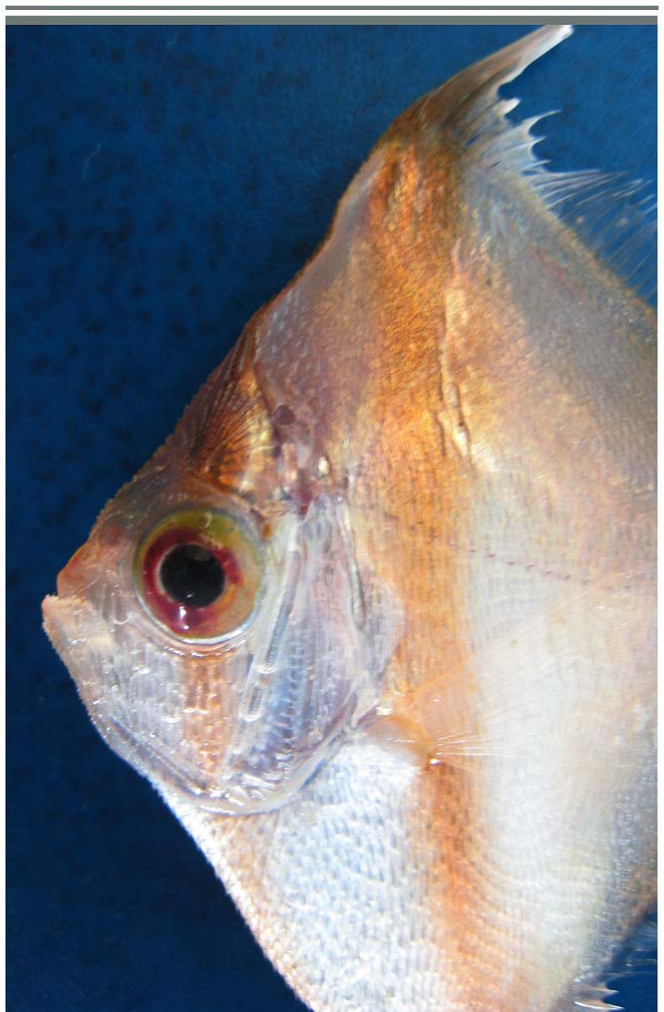
*Antigonia capros*, 37 mm SL, close-up of head, photo by W Toller.

*Antigonia capros* Lowe, 1843*—*deepbody boarfish; **USNM, F**; Figure 187

*Antigonia combatia* Berry & Rathjen 1959*—*shortspine boarfish; **UF, T**

The shortspine boarfish record from Saba Bank is based on a trawl collection with vouchered museum specimens at UF.

### Bothidae—lefteye flounders

*Bothus lunatus* (Linnaeus, 1758)*—*peacock flounder; **O**

**Figure 188:**
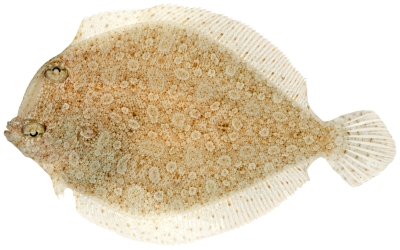
*Bothus ocellatus*, 95.8 mm SL, photo by JT Williams.

*Bothus ocellatus* (Agassiz, 1831)*—*eyed flounder; **UF**, **USNM, I**; Figure 188

*Trichopsetta ventralis* (Goode & Bean, 1885)*—*sash flounder; **UF, T**

### Paralichthyidae—sand flounders

*Citharicthys dinoceros* Goode & Bean, 1886*—*spined whiff; **USNM, T**

### Cynoglossidae—tonguefishes

**Figure 189:**
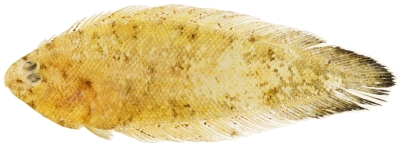
*Symphurus arawak*, 30.7 mm SL, photo by JT Williams.

*Symphurus arawak* Robins & Randall, 1965*—*Caribbean tonguefish; **USNM, I**; Figure 189

**Figure 190:**
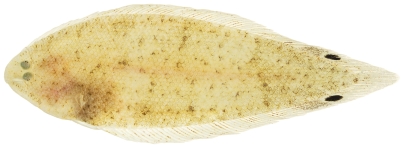
*Symphurus ommaspilus*, 42.9 mm SL, photo by JT Williams.

*Symphurus ommaspilus* Böhlke, 1961*—*ocellated tonguefish; **USNM, I**; Figure 190

### Balistidae—triggerfishes

*Balistes vetula* Linnaeus, 1758*—*queen triggerfish; **USNM, O, V**

*Canthidermis sufflamen* (Mitchill, 1815)*—*ocean triggerfish; **O, V**

*Melichthys niger* (Bloch, 1786)*—*black durgon; **USNM, O, V**

**Figure 191:**
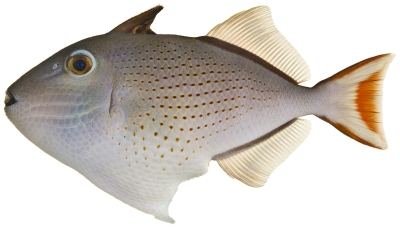
*Xanthichthys ringens*, 87.3 mm SL, photo by JT Williams.

*Xanthichthys ringens* (Linnaeus, 1758)*—*sargassum triggerfish; **USNM, I**; Figure 191

### Monacanthidae—filefishes

*Aluterus scriptus* (Osbeck, 1765)*—*scrawled filefish; **USNM, F, V**

**Figure 192:**
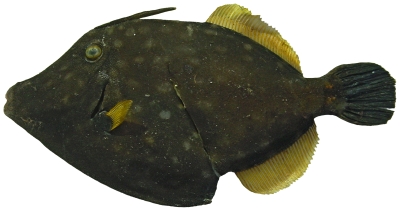
*Cantherhines macrocerus*, 273 mm SL, image flipped horizontally – right side is shown, photo by W Toller.

*Cantherhines macrocerus* (Hollard, 1853)*—*whitespotted filefish; **USNM, F**; Figure 192

*Cantherhines pullus* (Ranzani, 1842)*—*orangespotted filefish; **O, V**

**Figure 193:**
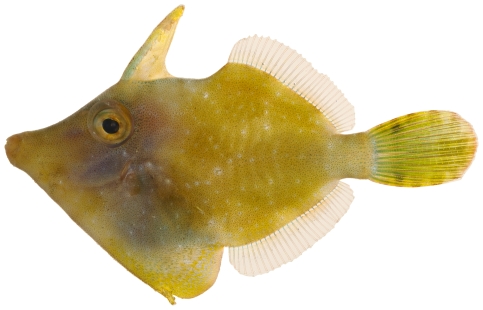
*Monacanthus ciliatus*, 33.4 mm SL, photo by JT Williams.

*Monacanthus ciliatus* (Mitchill, 1818)*—*fringed filefish; **USNM, I, V**; Figure 193

**Figure 194:**
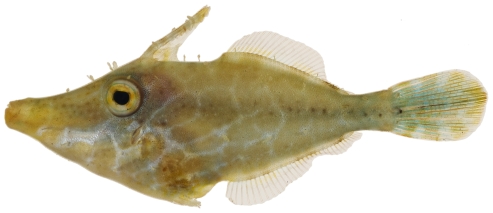
*Monacanthus tuckeri*, 32.3 mm SL, photo by JT Williams.

*Monacanthus tuckeri* Bean, 1906*—*slender filefish; **USNM, I**; Figure 194

### Ostraciidae—boxfishes

*Acanthostracion polygonia* Poey, 1876*—*honeycomb cowfish; **USNM, F, O, V**

*Acanthostracion quadricornis* (Linnaeus, 1758)*—*scrawled cowfish; **USNM, F, O**

*Lactophrys bicaudalis* (Linnaeus, 1758)*—*spotted trunkfish; **F**

**Figure 195:**
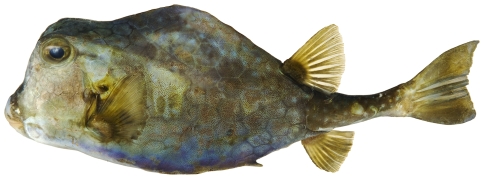
*Lactophrys trigonus*, 293.1 mm SL, photo by JT Williams.

*Lactophrys trigonus* (Linnaeus, 1758)*—*trunkfish; **USNM, I, V**; Figure 195

**Figure 196:**
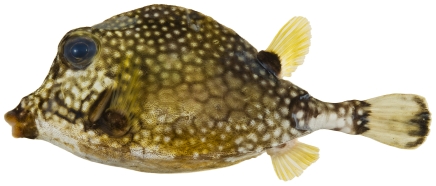
*Lactophrys triqueter*, 111.7 mm SL, photo by JT Williams.

*Lactophrys triqueter* (Linnaeus, 1758)*—*smooth trunkfish; **USNM, F, OBS, VIS**; Figure 196

### Tetraodontidae—puffers

**Figure 197:**
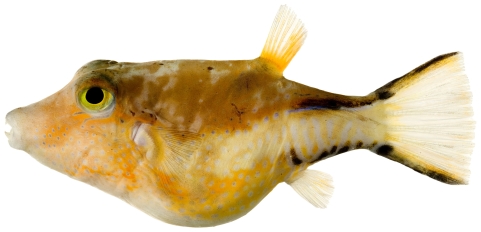
*Canthigaster rostrata*, 50.9 mm SL, photo by JT Williams.

*Canthigaster rostrata* (Bloch, 1786)*—*sharpnose puffer; **USNM, I, O, V**; Figure 197

**Figure 198:**
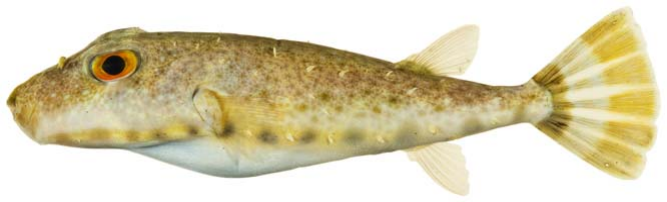
*Sphoeroides spengleri*, 73.1 mm SL, photo by JT Williams.

*Sphoeroides spengleri* (Bloch, 1785)*—*bandtail puffer; **USNM, I, O**; Figure 198

### Diodontidae—porcupinefishes

**Figure 199:**
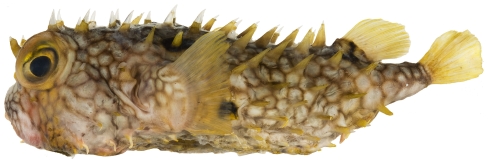
*Chilomycterus antillarum*, 180.0 mm SL, photo by JT Williams.

*Chilomycterus antillarum* Jordan & Rutter, 1897*—*web burrfish; **USNM, F**; Figure 199

**Figure 200:**
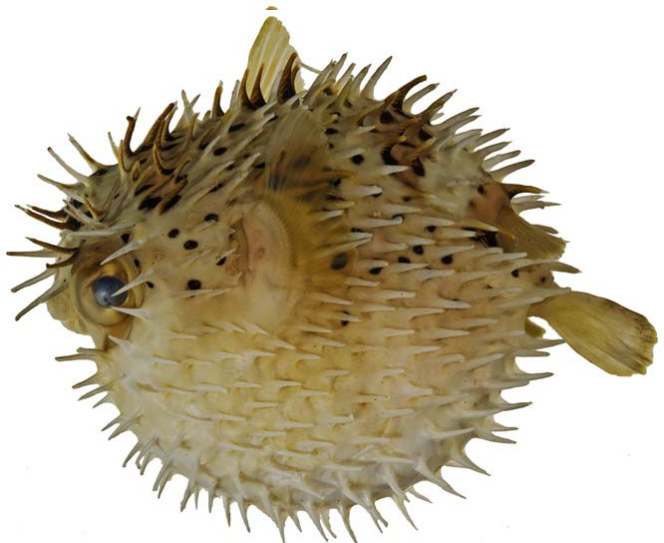
*Diodon holocanthus*, 104.9 mm SL, photo by JT Williams.

*Diodon holocanthus* Linnaeus, 1758*—*balloonfish; **USNM, I, V**; Figure 200

*Diodon hystrix* Linnaeus, 1758*—*porcupinefish; **O**

## Discussion

We document the occurrence of 270 species of fishes at Saba Bank. The diversity of fishes at Saba Bank is comparable (Table 1) to that of the oceanic atolls of Colombia (273 species), the islands in the Mona Passage of Puerto Rico (261 species) and Buck Island Reef National Monument (BIRNM; 262 species). The relatively high diversity of fishes at Saba Bank exists despite the lack of emergent land at the bank. There is no shallow-water shore-fish fauna represented on the bank due to the absence of a high-energy shoreline. These habitats typically add significantly to the fish diversity of Caribbean habitats. For example, the tube blennies (family Chaenopsidae) are a group of shorefishes typically found in fairly shallow coastal waters. According to Williams [4], there are approximately 22 recognized species of tube blennies known to occur in the central Caribbean, but only three of these species were found on Saba Bank. In addition, the absence of mangrove vegetation and apparent lack of sea-grass beds also limits the fish fauna of Saba Bank. In their quantitative study of a number of Saba Bank habitats, Toller et al. [5] attribute the apparent lack or rarity of a number of fish species found on Saba Bank to the absence of those habitats as nurseries for the juvenile stages of these fish species. Nevertheless, diverse habitat types exist on Saba Bank ranging from coral reefs and algal flats to soft-bottom lagoon areas and scoured hard, flat, pavement-like zones. These diverse habitats support a highly diverse, fish fauna and include a number of undescribed, new species along with species rarely encountered elsewhere in the Caribbean.

**Table 1:** Number of fishes recorded at well sampled sites in the Greater Caribbean.

Site	No. fish species	Source
Alligator Reef, FL	517	Starck [12]
Dry Tortugas, FL	442	Longley & Hildebrand [13]
Bermuda	433	Smith-Vaniz et al [14]
St. Croix	400	Clavijo et al [15]
Barrier Reef, Belize	339	C.L. Smith et al [9]
Offshore Banks, Belize	293	C.L. Smith et al [9]
Oceanic Atolls, Colombia	273	Mejia et al [16]
Saba Bank	270	This paper
Buck Island Reef	262	Smith-Vaniz et al [8]
Mona Passage Islands, Puerto Rico	261	Dennis et al [7]
Flower Garden Bank Nat. Mar. Sanctuary, Texas, USA	240	E. Hickerson, NOAA, pers. comm., Dec. 2009
Navassa Island, USA	237	Collette et al [6]
Rhomboidal Cays, Belize	193	C.L. Smith et al [9]
Core Pelican Cays, Belize	168	C.L. Smith et al [9]
Peripheral Rhomboidal Cays, Belize	123	C.L. Smith et al [9]

**Figure 201:**
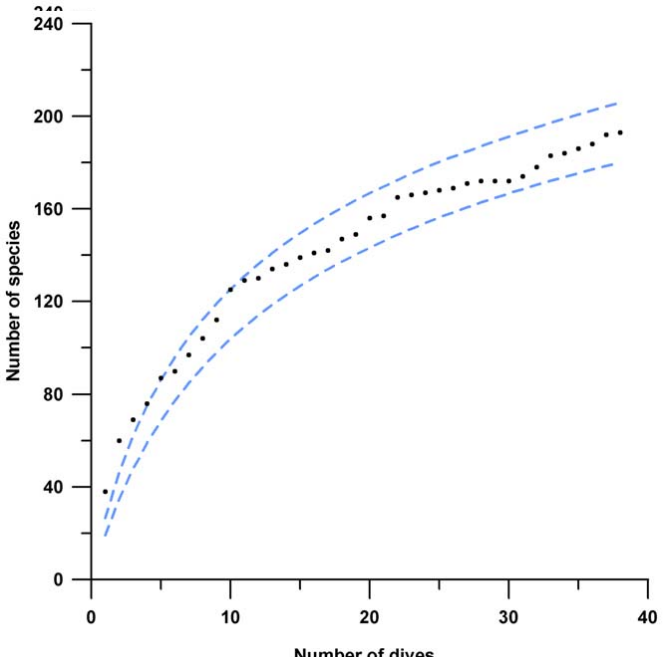
Actual species-accrual curve (black dots) for 38 dives collecting and identifying fish species on Saba Bank. Sobs (Mao Tau) 95% confidence intervals [8] are shown as light blue dashed lines.

The highly diverse, fish fauna so far reported appears to substantially under represent the species richness of fishes for Saba Bank. The actual species-accumulation curve of rotenone collections and visual surveys combined do not reach an asymptote (Figure 201). The expected species-accumulation curves (Chao2 and Jack1 estimators in EstimateS) predict total fish-species richness somewhere between 320 and 411 species.

Of the 270 species we report (Table 1) from Saba Bank, 132 (49%) were observed during visual surveys. This result is comparable to the findings for Navassa Island where 41% of the fish species were detected by visual surveys [6], for the Mona Passage islands with 43% of the fish species detected visually [7], and for BIRNM with 44% of the fish species detected visually [8]. A higher percentage of fish species was detected visually at Saba Bank than at Navassa, Mona, or BIRNM. This could possibly be a result of the higher number of visual surveys carried out at Saba Bank than at Navassa or Mona. Although the BIRNM results are based on a higher number of visual surveys (70), a comparable percentage of the fauna was detected visually at BIRNM. BIRNM ichthyocide surveys (58) appear to demonstrate the ability of ichthyocide collections to more thoroughly sample the fauna (see below). Visual censuses have numerous biases as discussed in the methods for the Pelican Cays study [9]. Despite the slight differences in methods and sampling designs between the published fish species-richness studies in different parts of the Caribbean, the methods utilized produce consistent and comparable results.

Ichthyocide collections at Saba Bank yielded specimens representing 155 fish species (57% of the total fish fauna). At Navassa Island where there were fewer visual surveys than at Saba Bank, over 70% of the fish species were collected with ichthyocide [6], at the Mona Passage islands, 61% of the fish species were collected with ichthyocide [7], and at BIRNM, 87% of the fish species were collected with ichthyocide despite their being more visual surveys in the BIRNM study [8]. By occupying 58 (27 at Saba Bank) ichthyocide stations covering a broader diversity of ecological habitats, the BIRNM study appears to have obtained a more thorough sampling of the resident species. The lower number of species taken at Saba Bank with ichthyocide is at least partially due to the absence of shallow habitat. Shallow habitat is required by many of the small cryptic fish species that are normally detected only with the use of ichthyocide and these species have not been found at Saba Bank. The prevalence of strong currents across Saba Bank further limits the effectiveness of ichthyocide, which is only effective when relatively high concentrations of ichthyocide remain in one place (preferably in a confined area) for more than 15 to 20 minutes. The ichthyocide-collecting methods employed at Saba Bank were the same as those used at Navassa Island and Belize. The BIRNM ichthyocide collecting methods differed from ours only in that the BIRNM study used a block net in addition to collecting outside the net. Nevertheless, the BIRNM methods were comparable to ours because the BIRNM study included those species collected outside the block nets in their results. There are inherent biases in ichthyocide collecting due to unpredictability of environmental parameters, such as currents, temperature, degree of confinement of the sampling area, ability of larger species to swim away, and variable assays of active rotenone in the powdered *Derris* root (batches often vary from 5% to over 11% active rotenone due to natural variation in rotenone concentration among the roots of different plants). Despite these biases, ichthyocide collections yield comparable results as described above.

Neither visual surveying nor ichthyocide collecting alone are capable of providing a comprehensive survey of coastal (or submerged atoll in the case of Saba Bank) fish species. A combination of visual surveys, ichthyocide sampling using SCUBA, and various fishing techniques must be employed to effectively assess fish-species richness in marine habitats shallow enough to be accessible to SCUBA divers.

The number of fish species living on Saba Bank is undoubtedly higher than 270 as indicated by Chao2 and Jack1 estimators. As most parts of Saba Bank are deeper than 25 m, sampling with ichthyocides using SCUBA is limited by the reduced bottom time at these depths. As a result we focused primarily on the rim (shallowest parts) of the submerged atoll to maximize bottom time for collecting. Future sampling with ichthyocides applied by divers utilizing rebreathers, supplemented with trawl and dredge sampling would allow collecting from the outer slopes and would certainly yield additional new and interesting species of fishes from this submerged atoll.

## Methods

The NMNH/SI Animal Care and Use Committee approved the methods and procedures utilized during the course of this biodiversity assessment project. All Saba Bank projects had collecting permits through the Convention on Trade in Endangered Species (CITES, where necessary) and the Saba Conservation Foundation (where CITES was not required).

Roving surveys were completed using SCUBA and lasted 60 minutes, bottom time permitting. All species encountered were listed on a slate while swimming in a haphazard pattern covering all bottom depths possible down to a maximum of 38 m. Other visual surveys are described in Toller et al [5]. Collecting methods follow Collette et al [6]. Species were photographed in aquaria after the fins were pinned out and brushed with formaldehyde solution. Tissue samples were taken from fresh specimens and the voucher specimens were preserved in a formaldehyde solution diluted with water to 3.75% formaldehyde. Large specimens were also injected with 37.5% formaldehyde before being soaked in the 3.75% formaldehyde solution.

After arrival at the Museum Support Center (MSC), National Museum of Natural History, specimens were transferred sequentially through water-diluted solutions of 25% ethanol, 50% ethanol, and finally into 75% ethanol for permanent archival storage. Specimens were then processed and cataloged into the USNM at the MSC in Suitland, MD.

To generate species-accumulation curves, a data matrix of presence/absence was constructed from 38 combined roving surveys, rotenone collections, and fish-habitat transects using species as variables and dives as observations (Table 2 provides details for each survey and collecting station). The matrix was employed for actual and expected species-accumulation curves (Mao Tau) in EstimateS v.8.0 software [10]. Total expected richness was estimated using Chao2 and Jack1 estimators.

**Table 2:** Collecting stations occupied on Saba Bank, 2006-2007.

Station number	Roving & Rotenone	Coordinates	Date	Depth (m)	Local name	Description of habitat
Saba-06-01	RR01	17o28.778N; 63o13.663W	04 Jan 2006	30	North East Reef	N.E. reefs, just S of Poison Bank. Spur and groove reef, low relief, many algae (Dictyota)-rotenoned a groove in reef with sand at bottom, walls of groove with low corals
Saba-06-02	RR02	17o26.883N; 63o54.055W	05 Jan 2006	38	Small Bank South	Small Bank South. Scattered corals, many *Xestospongia*. Nassau grouper, yellowfin grouper, and black grouper sighted. Nurse shark-rotenoned area with encrusting corals
Saba-06-03	RR03	17o25.778N; 63o41.037W	05 Jan 2006	35	Rhodolith Reef	Western patch reef, many rodoliths-rotenoned a flat area with sponges and rubble bottom
Saba-06-04		17o18.557N; 63o22.019W	05 Jan 2006	35		Fish Trap- Local Fishermen
Saba-06-05	RR04	17o24.602N; 63o11.748W	06 Jan 2006	27	Redman Bulge	Eastern reef, spur and groove, medium height, many algae (dictyota)
Saba-06-06	RR05	17o26.028N; 63o16.536W	06 Jan 2006	22	Seaweed city	pavement covered with ca. 10 cm sand layer, *Pseudopterogorgia*, many algae species
Saba-06-07		17o33.697N; 63o46.949W		0-1		Caught on hook and line while trolling over Saba Bank
Saba-06-08	RR06	17o33.092N; 63o28.758W	07 Jan 2006	37	Grouper Bank	bare pavement, occasional solution holes, Manicinias, many conch (old)
Saba-06-09	RR07	17o34.893N; 63o24.400W	07 Jan 2006	30	Rendezvous Hill	pavement with corals, low relief-rotenoned a low-relief coral reef with soft and hard corals, and sponges
Saba-06-10		17o25.883N; 63o21.874W	08 Jan 2006	0-1		Caught on hook and line while trolling over Saba Bank
Saba-06-11	RR08	17o14.070N; 63o26.915W	08 Jan 2006	25	Butterfly Reef	Southern outer reef. Spur and groove, medium height (2-3 ft)
Saba-06-12	RR09	17o14.380N; 63o26.915W	08 Jan 2006	18	Brain coral reef	Southern inner reef, rock pavement, scattered corals
Saba-06-13	RR10	17o33.801N; 63o17.806W	09 Jan 2006	32	Fishpot surprise	sloping pavement with ledges, sand patches and large rubble, algae, scattered corals-rotenoned an area with sand and loose rock at base of rocky slope with brown algae abundant.
Saba-06-14		17o33.970N; 63o17.227W	09 Jan 2006	40		Fish Trap
Saba-06-15		17o33.897N; 63o17.730W	09 Jan 2006	40		Fish Trap
Saba-06-16		17o33.878N; 63o17.168W	10 Jan 2006	40		Fish Trap
Saba-06-17	RR11	17o33.849N; 63o17.872W	10 Jan 2006	26	Lost anchor	Sloping pavement, patches of large rubble, algae, scattered corals; red hind spawning area
Saba-06-18		17o33.837N; 63o17.962W	10 Jan 2006	30		Fish Trap
Saba-06-19	RR12	17o33.686N; 63o17.629W	10 Jan 2006	26	Moonfish Bank	Sloping pavement, sand patches, large rubble, algae, scattered corals; red hind spawning area
Saba-06-20		17o30.751N; 63o13.632W	12 Jan 2006	29	Poison Bank	Rotenone
Saba-06-21		17o28.046N; 63o14.978W	12 Jan 2006	19		Rotenone-northeastern shallow flats
Saba-06-22		17o26.390N; 63o27.776W	13 Jan 2006	26		Rotenone-flat bottom in central area of bank
Saba-06-23		17o30.580N; 63o27.595W	13 Jan 2006	28		Rotenone-flat bottom in central area of bank
Saba-06-24		17o20.766N; 63o15.008W	14 Jan 2006	31	Coral Garden	Rotenone-coral groove at top of steep outer dropoff on SE side of Saba Bank, rock, coral, and sponges, sand at bottom of groove.
Saba-06-25		17o21.162N; 63o15.138W	14 Jan 2006	18		Rotenone-near Coral Garden at SE edge of Saba Bank, small ledge on edge of sand and rubble flat, gorgonians, coral and rock
Saba-06-26						No label with these-specimens taken at one or more of the other stations, but their locality labels were lost
Saba 2007-01		17.51206 N; 63.2332 W	20 Jun 2007	29-38		Poison Bank- rotenone-dead and live coral channel with rubble on deep reef.
Saba 2007-02		17.46028 N; 63.2517 W	20 Jun 2007	15-19		Twin Peaks- rotenone-algae covering rock and some sand on a low ridge

**Figure 202:**
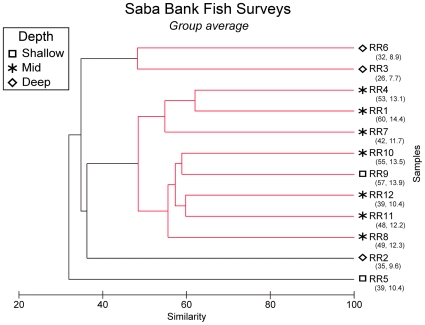
Hierarchical clustering dendrogram of stations showing significant differences (black bars) among fish assemblages at deep stations versus middle-depth and shallow stations (SIMPROF, *P*<0.05).

A Bray-Curtis resemblance matrix was generated from a matrix of presence/absence data from 12 combined roving surveys and rotenone collections in order to produce a non-metric multidimensional scaling (MDS) ordination and group averaged hierarchical clustering dendrogram in PRIMER v. 6.1 software [11]. The ANOSIM statistic was employed to test for *a priori* differences between habitat types and depth classes. A dendrogram based on group averaged hierarchical cluster techniques was used to illustrate the differences among depth classes (Figure 202). Color codes are derived from the similarity profile (SIMPROF) statistic in Primer 6.1. SIMPROF is less powerful than ANOSIM, intended for *a posteriori* tests of structure in the data. The test was employed here for representational purposes. A second data matrix of species presence and absence was introduced for an *a posteriori* test of genuine data structure among Caribbean localities, using the Bray-Curtis resemblance measure to determine the level of similarity among localities. Principal Components Analysis (PCA) was also conducted in PRIMER 6.1 in order to determine the fish species primarily responsible for differences among habitats.
