# Correction: Biodiversity Assessment of the Fishes of Saba Bank Atoll, Netherlands Antilles

**DOI:** 10.1371/annotation/c9e7d37a-0514-4aa7-affb-44d206453530

**Published:** 2010-06-24

**Authors:** Jeffrey T. Williams, Kent E. Carpenter, James L. Van Tassell, Paul Hoetjes, Wes Toller, Peter Etnoyer, Michael Smith

The species name of the organism shown in Figure 68 is incorrect. The correct species name is *Heteropriacanthus cruentatus*. 

